# An integrated genomic approach identifies persistent tumor suppressive effects of transforming growth factor-β in human breast cancer

**DOI:** 10.1186/bcr3668

**Published:** 2014-06-02

**Authors:** Misako Sato, Mitsutaka Kadota, Binwu Tang, Howard H Yang, Yu-an Yang, Mengge Shan, Jia Weng, Michael A Welsh, Kathleen C Flanders, Yoshiko Nagano, Aleksandra M Michalowski, Robert J Clifford, Maxwell P Lee, Lalage M Wakefield

**Affiliations:** 1 Laboratory of Cancer Biology and Genetics, Center for Cancer Research, National Cancer Institute, 37 Convent Drive, Bethesda, MD 20892, USA; 2 Laboratory of Population Genetics, Center for Cancer Research, National Cancer Institute, 41 Library Drive, Bethesda, MD 20892, USA; 3 Department of Hepatology, Graduate School of Medicine, Osaka City University, 1-4-3 Asahimachi, Abeno, Osaka 545-8585, Japan; 4 Genome Resource and Analysis Unit, RIKEN Center for Developmental Biology, 2-2-3 Minatojima-minami-machi, Chuo-ku Kobe, Hyogo 650-0047, Japan; 5 Department of Immunotherapeutics, Tokyo Medical and Dental University, 1-5-45 Yushima, Bunkyo-ku, Tokyo 113-8519, Japan; 6 Walter Reed Army Institute of Research, 503 Robert Grant Avenue, Silver Spring MD 2091, 0USA

## Abstract

**Introduction:**

Transforming growth factor-βs (TGF-βs) play a dual role in breast cancer, with context-dependent tumor-suppressive or pro-oncogenic effects. TGF-β antagonists are showing promise in early-phase clinical oncology trials to neutralize the pro-oncogenic effects. However, there is currently no way to determine whether the tumor-suppressive effects of TGF-β are still active in human breast tumors at the time of surgery and treatment, a situation that could lead to adverse therapeutic responses.

**Methods:**

Using a breast cancer progression model that exemplifies the dual role of TGF-β, promoter-wide chromatin immunoprecipitation and transcriptomic approaches were applied to identify a core set of TGF-β-regulated genes that specifically reflect only the tumor-suppressor arm of the pathway. The clinical significance of this signature and the underlying biology were investigated using bioinformatic analyses in clinical breast cancer datasets, and knockdown validation approaches in tumor xenografts.

**Results:**

TGF-β-driven tumor suppression was highly dependent on Smad3, and Smad3 target genes that were specifically enriched for involvement in tumor suppression were identified. Patterns of Smad3 binding reflected the preexisting active chromatin landscape, and target genes were frequently regulated in opposite directions *in vitro* and *in vivo,* highlighting the strong contextuality of TGF-β action. An *in vivo-*weighted TGF-β/Smad3 tumor-suppressor signature was associated with good outcome in estrogen receptor-positive breast cancer cohorts. TGF-β/Smad3 effects on cell proliferation, differentiation and ephrin signaling contributed to the observed tumor suppression.

**Conclusions:**

Tumor-suppressive effects of TGF-β persist in some breast cancer patients at the time of surgery and affect clinical outcome. Carefully tailored *in vitro/in vivo* genomic approaches can identify such patients for exclusion from treatment with TGF-β antagonists.

## Introduction

Gene expression profiling approaches have highlighted the molecular heterogeneity of breast cancer [1], and identified gene expression fingerprints of molecular pathway activation [2]. A greater understanding of the contribution of different signaling pathways will be critical for the development of precision medicine approaches to cancer therapy. Transforming growth factor-βs (TGF-βs) are highly pleiotropic regulatory proteins that play complex roles in epithelial carcinogenesis, and the prevailing dogma is that they switch from a predominantly tumor-suppressive role to a tumor-promoting role as disease progresses (reviewed in [3-5]). Based on encouraging preclinical data showing that the deleterious pro-oncogenic arm of the TGF-β biological response program can be effectively blockaded for therapeutic benefit, TGF-β antagonists are now in early phase clinical trials in oncology in several tumor types [6], including breast cancer (http://clinicaltrials.gov Trial NCT01401062). However, the specter remains that such interventions could inadvertently interfere with residual tumor suppressive activity and thus adversely affect outcome. Here we have asked if genomic approaches can be used to discern whether tumor-suppressive effects of TGF-β do indeed persist and influence survival in any human breast cancers at the time of surgery.

Mechanisms underlying the dual role model for TGF-β in cancer progression involve a wide variety of TGF-β effects on both the tumor parenchyma and the supporting stromal microenvironment. Tumor-suppressive effects include the induction of various protective responses to counteract genetic damage and oncogene activation [7-10], as well as the maintenance of a tumor-suppressive cytokine and chemokine profile in the microenvironment [11-13]. However as disease progresses, activation of oncogenic pathways in the tumor parenchyma can not only override the tumor-suppressive responses to TGF-β, but can also unmask pro-progression responses such as induction of the epithelial-to-mesenchymal transition, enhanced migration and invasion, and expansion of the cancer stem cell compartment [14-18]. At the same time, the excessive TGF-β that is frequently found in the microenvironment of advanced tumors can subvert antitumor immune surveillance, promote angiogenesis, and generally contribute to the development of a more supportive tumor stroma [19-21].

Preclinical studies in model systems have provided considerable support for a dual role for TGF-β in breast cancer (reviewed in [22,23]). TGF-β was shown to switch from tumor-suppressor to prometastatic factor with disease progression in both a HER2/Neu-driven genetically engineered mouse model and a Ras-driven human xenograft model of breast cancer [24,25]. In contrast, studies in the MMTV-PyVT mouse model of breast cancer have suggested that tumor-suppressive effects of the TGF-β pathway may persist even in late-stage metastatic disease [26-28]. Currently, the relative importance of the two different aspects of TGF-β biology in determining clinical outcome in human breast cancer patients is not clear. TGF-β pathway components are rarely mutated or deleted in breast cancer [29], so the effects of any TGF-β pathway perturbation in the clinical situation are likely to be more subtle than is seen with preclinical knockout models. Interestingly, the majority of human breast cancer cell lines have lost their growth inhibitory responses to TGF-β *in vitro*[30], and only MCF10Ca1h cells have been definitively shown to retain tumor-suppressive responses to endogenous TGF-β *in vivo*[25]. This situation could reflect an early loss of TGF-β-driven tumor suppression in the majority of human breast cancers. Indeed, reduced expression of the type II TGF-β receptor has been seen in epithelial hyperplasia without atypia, the very earliest preneoplastic lesion of the breast [31]. Alternatively, it may simply reflect challenges in establishing cell lines from breast cancers that retain such responses.

The possible persistence of tumor-suppressive effects of TGF-β in human breast cancers at the time of clinical intervention has profoundly important implications for the deployment of TGF-β-targeted therapies. To address this question rigorously, we chose to develop a gene signature for TGF-β-driven tumor suppression. Several TGF-β-related gene expression signatures have been developed previously [2,11,18,32-36], but they were not designed *a priori* to discriminate between tumor-suppressive and pro-progression responses to TGF-β. Furthermore, such signatures are almost invariably associated with poor prognosis, suggesting that the pro-oncogenic activities of the TGF-β pathway are more readily captured by these approaches than are the tumor-suppressive activities. Here we describe an integrated *in vitro*/*in vivo* genomic strategy to identify a gene signature that specifically reflects TGF-β-driven tumor-suppressive effects on the tumor parenchyma. We show that high expression of the signature predicts good outcome in clinical datasets from estrogen receptor-positive (ER+) breast cancer patients, a finding that suggests there is a subset of such patients who should not be treated with TGF-β pathway antagonists. Our approach also revealed novel aspects of TGF-β biology, highlighting effects of TGF-β on breast cancer differentiation and linking ephrin signaling to TGF-β-mediated tumor suppression.

## Methods

### Cell lines and reagents

The MCF10A-derived cell lines [37,38] were obtained from the Barbara Ann Karmanos Cancer Institute Cell Line Resource, (Detroit, MI, USA) and were cultured as previously described [25]. MCF10A (‘M1’) cells are spontaneously immortalized human breast epithelial cells derived from a woman with fibrocystic breast disease [39]. MCF10AT1k.cl2 (‘M2’) was derived from MCF10A by transfection with mutant Ha-Ras followed by *in vivo* selection for cells that gave rise to preneoplastic lesions and subsequent *in vitro* cloning from the lesions. MCF10Ca1h (‘M3’) and MCF10Ca1a.cl1 (‘M4’) were cultured from rare tumors that arose from the premalignant MCF10AT1k.cl2 cells following implantation *in vivo*[37,38]. Although the MCF10A parental cell line is ER-negative, the derivative Ras-transformed cell lines show varying degrees of ER positivity and biological responses to estrogen *in vitro* and *in vivo*[40-43]. In particular, we and others have shown that tumors derived from M3 cells contain ER+ cells in the differentiated regions ([38] and Additional file 1) and thus model ER+ breast cancer. They are genetically wild type for p53 but have mutated PIK3CA [44], which are also characteristics of human ER+ breast cancers [45,46]. Smad2 and Smad3 null conditionally immortalized mouse mammary epithelial cells (IMECs) were generated and cultured as described previously [47]. For all *in vitro* assays, cells were grown to 50 to 60% confluence and then serum-starved in DMEM/F12 supplemented with Serum Replacement 1 (Sigma-Aldrich, St. Louis, MO, USA) for 16 hours prior to TGF-β treatment. Recombinant human EphrinA1-Fc chimera was purchased from R&D Systems Inc. (Minneapolis, MN, USA).

### ShRNA knockdown and gene overexpression

For gene knockdown experiments, short hairpin RNA (shRNA) against target sequences in SMAD2 (5′-GCACTTGCTCTGAAATTTG-3′) and SMAD3 (5′-GGCCATCACCACGCAG AAC-3′), were cloned into the pLKO.1 lentiviral vector. shRNA against GFP RNA (5′-AAGACCCGCGCCGAGGTGAAG-3′) was used as a control. EFNA1 shRNA lentiviral constructs (TRCN0000007311 in pLKO.1; V3LHS_360202, V3LHS_360203 in pGIPZ) were purchased from Open Biosystems Inc., (Huntsville, AL, USA). A dominant negative human type II TGF-β receptor (dnTβRII) consisting of amino acids 1-219 of TGFBR2 coupled to a Myc or V5 tag was cloned into pLPCX retroviral (BD Biosciences Clontech, Palo Alto, CA, USA) or pLenti6.2/V5-DEST Gateway lentiviral (Invitrogen, Carlsbad, CA, USA) backbones respectively. Lentiviral constructs were transfected into the 293FT producer cell line using the pPACKH1 lentivector packaging kit (System Biosciences, Mountain View, CA, USA) and Lipofectamine 2000 (Invitrogen). Pseudoviral particles were isolated by centrifugation and incubated with M3 and M4 cells for 24 hours. The cells were grown for an additional 48 hours after transduction and then maintained under puromycin selection (4 μg/ml) for five days. *In vivo* experiments were performed within three to five passages after transduction using pools of transduced cells. Knockdown or overexpression were confirmed by RT-QPCR and Western blot analysis. Activity of the dnTβRII was confirmed by the ability to block TGF-β-inducible Smad2 phosphorylation and TGF-β-inducible activity of the Smad3 reporter CAGA12-luciferase, following transient transfection with pGL3(CAGA12)-Luc.

### Western blotting

Whole-cell extracts were prepared in M-PER (Thermo Fisher Scientific Inc., Pittsburgh, PA, USA) containing Complete Protease Inhibitor cocktail (Roche Applied Science, Indianapolis, IN, USA) and phenylmethylsulfonyl fluoride (Sigma-Aldrich). Typically, 35 μg of total protein was loaded on to 4 to 20% Tris-Glycine SDS-PAGE gels (Invitrogen) and transferred to PVDF membrane (Millipore, Temecula, CA, USA). Antibodies were as follows: Smad3 antibody (Ab28379, ChIP grade, Abcam, Cambridge, MA, USA), Smad2 antibody (15-1300, Invitrogen), anti-Smad3 phosphoS423/425 antibody (Smad3-CP, 1880-1, Epitomics, Burlingame, CA, USA), anti-linker phospho S208 Smad3 antibody (Smad3-LP) was generously provided by Dr. Fang Liu (Rutgers University, NJ, USA), anti-Ephrin-A1 antibody (3880-1, Epitomics or SC-911, Santa Cruz Biotechnology Inc., Santa Cruz, CA, USA), anti-Eck/EphA2, clone7 (05-480, Millipore), anti-phosphoEphA2-S897 (035118, US Biological, Salem, MA, USA). Anti-β-actin (A-2228, Sigma-Aldrich) was used to assess equivalence of protein loading. Peroxidase-conjugated secondary antibodies were used at 1:5000 dilution and the signals were detected by ECL (Thermo Scientific Pierce, Rockford, IL, USA). Scanned Western blots were quantitated using MultiGauge Analysis Software (Fujifilm, Tokyo, Japan).

### Chromatin immunoprecipitation (ChIP) and methylated DNA immunoprecipitation (MeDIP)

Cells were grown to 50 to 60% confluence in complete medium and then switched to DMEM/F12 medium containing Serum Replacement 1 (Sigma-Aldrich) at a final concentration of 1x for 16 hours before treatment with 5 ng/ml of TGF-β1 or vehicle for 1 hour. 1 × 10^8^ cells from vehicle or TGF-β-treated cultures were washed once with PBS and then dual cross-linked at room temperature successively with 2 mM di-(*N*-succinimidyl) glutarate (DSG, Thermo Scientific) for 30 minutes and 1% formaldehyde for 10 minutes. We found the DSG cross-linking step significantly increased the recovery of TGF-β-induced Smad3-bound DNA when compared with formaldehyde fixation alone. Glycine was added to a final concentration of 0.25 M to stop the fixation. Cells were washed twice with ice-cold PBS, scraped, snap-frozen in liquid nitrogen and stored at -80C until further processing. For Smad3 ChIP, cells were resuspended and sonicated in 3 ml of SDS lysis buffer (1% SDS, 10 mM EDTA, 50 mM Tris-HCl, pH 8.0) to a fragment size of 200 to 500 bp on ice using a Misonix™ sonicator with the following pulse parameters: time on = 15 seconds, time off = 10 seconds, total sonication time for 10 minutes at power level = 3 to 4. The lysates were centrifuged at 15,000 × g for 10 minutes and diluted 10-fold with ChIP dilution buffer (0.01% SDS, 16.7 mM Tris-HCl, pH 8.0, 1.2 mM EDTA, 1.1% Triton™ X-100, 167 mM NaCl) and precleared with Dynabead™ Protein A (Invitrogen) for 30 minutes, followed by incubation with 3 μg/ml anti-Smad3 antibody (# 28379, ChIP grade, Abcam) or rabbit immunoglobulin G (IgG) for overnight at 4°C. Protein A beads were added and incubated for 1 hour at 4°C, and beads were then washed successively with low (0.1% SDS, 20 mM Tris-HCl, pH 8.0, 2 mM EDTA, 1% Triton™ X-100, 150 mM NaCl) and high (0.1% SDS, 20 mM Tris-HCl, pH 8.0, 2 mM EDTA, 1% Triton™ X-100, 500 mM NaCl) salt washing buffer, LiCl wash buffer (10 mM Tris-HCl, pH 8.0, 1 mM EDTA, 1% NP-40, 1% deoxycholic acid sodium salt, 0.25 M LiCl) and TE buffer. ChIPed DNA was eluted in SDS elution buffer at room temperature (RT) with occasional vortexing and the cross-linking was reversed by overnight incubation at 65°C.

Smad3 ChIPed DNA was amplified using Whole Genome Amplification and Reamplification kits (Sigma-Aldrich). ChIP-QPCR for known Smad3 target genes (JUNB, SERPINE1, SMAD7) confirmed successful amplification. Amplified ChIPed DNA was biotinylated according to the standard Affymetrix protocol (Affymetrix Chromatin Immunoprecipitation Assay Protocol). Following fragmentation, 10 μg of biotinylated DNA was hybridized for 16 hours at 45°C to an Affymetrix promoter tiling array (GeneChip™ Human Promoter 1.0R Array; 25,500 promoters, 25-mer probes, Affymetrix, Santa Clara, CA, USA) according to the manufacturer’s instructions. Genechips™ were washed and stained in the Affymetrix Fluidics Station 450, and then scanned using an Affymetrix GeneChip™ Scanner 3000 7G. Data was collected using Affymetrix AGCC software. Each ChIP experiment was conducted in quadruplicate (M1 cells and M2 cells) or duplicate (M3 cells and M4 cells) for independent chromatin isolations.

Histone H3 with acetylation on lysine residues 9/14 (H3AcK9/14) has previously been shown to be highly localized to the 5′ regions of transcriptionally active human genes [48] or genes that are poised for transcription [49]. To determine the chromatin activation state of select Smad3 binding regions, ChIP analysis for acetylated histones in untreated M1 to M4 cells grown in complete medium was carried out as previously described [50], using a ChIP assay kit (Upstate, Temecula, CA, USA), with antibodies against H3AcK9/14 (antibody #06-599, Upstate). QPCR was performed to determine enrichment of target genomic regions in the immunoprecipitated fraction compared with input DNA. Similarly, to assess the methylation status of DNA targets, immunoprecipitation of methylated DNA (MeDIP) was performed. 1 μg whole DNA from all four cell lines (M1 to M4 untreated, complete medium) was sonicated in TE buffer (Sonicator, Misonix Inc., NY, USA; setting: power 3, 30 seconds ON, 20 seconds OFF, four times on ice). DNA samples were denatured at 99°C for 5 minutes then snap-cooled in iced water. Anti-5-methyl cytosine polyclonal antibody (CP51000, rabbit, Megabase Research Products, Lincoln, NE, USA) was incubated with the sonicated DNA for 2 hours at 4°C, followed by incubation with Dynabead Protein A (Invitrogen) for 1 hour. Supernatant was collected as unbound fraction, and beads were then washed three times with MeDIP washing buffer (10 mM Tris-HCl, 150 mM NaCl, 0.05% Triton X-100 and 0.01% BSA) and once with TE buffer. Immunoprecipitated DNA was eluted in SDS elution buffer (1% SDS, 10 mM Tris-HCl, pH 8.0, 5 mM EDTA, 300 mM NaCl), at RT for 10 minutes, and then incubated at 55°C for 3 hours with proteinase K. DNA was extracted using Qiagen Enzymatic reaction clean-up kit (Qiagen, Germantown, MD, USA) to give the MeDIP fraction. QPCR analysis was performed to determine enrichment of genomic regions in MeDIP fractions, normalized to unbound DNA.

### ChIP-QPCR and MeDIP QPCR

Smad3 occupancy at previously known targets as well as at select target genes identified by ChIP-chip was validated by QPCR of Smad3 ChIPed DNA following whole genome amplification, using Power SYBR Green PCR master mix (Applied Biosystems, Carlsbad, CA, USA) with ABI-PRISM 7900 Sequence Detection System (Applied Biosystems). The enrichment of ChIP DNA was calculated relative to the input DNA using gene-specific primer sets to the Smad binding region (SBRs) identified from the ChIP-chip analysis, and was compared with ChIP for control IgG. Similarly, QPCR of MeDIP DNA and of DNA ChIPed for histone H3AcK9/14 was performed for select SBRs. PPIA was used as a negative control for Smad3 ChIP and MeDIP and a positive control for H3AcK9/14 ChIP. Conversely, MyoD served as a positive control for MeDIP and a negative control for H3AcK9/14 ChIP. The primer pairs used for QPCR are given in Additional file 2.

### Smad3 ChIP-chip data analysis

Affymetrix Tiling Analysis Software (TAS, version 1.2.0) was used for data processing. Quantile normalization was performed within each comparison group; quadruplicate for M1 and M2 cells and duplicate for M3 and M4 cells. Signal or local *P* value were computed using a window size of 200 bp (bandwidth = 100 bp), and a minimum run of 200 bases with a maximum gap of 100 bases. Relative enrichment of TGF-β-dependent Smad3 binding was estimated from the signal difference between vehicle-treated and TGF-β-treated samples with a false discovery rate (FDR) of 15%. The analyzed data was visualized using Integrated Genome Brower [51] for each cell line. Enriched regions that overlapped with or were within 10 kb upstream or downstream of known promoter regions (Affymetrix Hs_PromP_NCBlv36.accession) were retained. Galaxy web server [52] and UCSC Genome Browser tables (‘hg18.knownGene’) were used to annotate enriched regions with target transcripts of known genes. Genes were assigned to bound genomic regions based on the gene transcription start site (TSS) distance to the midpoint of the Smad3 binding region (SBR). Both multiple target transcripts and the gene with the shortest TSS distance to the region midpoint were analyzed. On average, 72% of all enriched regions for each of the four cell lines were annotated with known genes.

To generate a probability density plot for the occurrence of SBRs in relation to the closest TSS, binding sites within the −7.5 kb to +2.5 kb genomic region for the target genes were used. Data for each cell line was analyzed independently. The distributions were obtained using kernel density estimates [53] implemented in R [54]. To determine the distribution of SMAD binding sites within the SBRs, a set of high confidence positive peaks identified in both M3 and M4 cells was used. After merging overlapping peaks, the final set had 190 regions. Sequences consisting of peak midpoints +/−1000 bp were divided in 100 bp bins. The bins of each peak were scored for the presence of one or more SMAD binding site. Data for bins equidistant from the midpoint (for example 100 bp upstream and 100 bp downstream) were combined. The fraction of bins with SMAD sites was compared at equivalent bin positions for the 190 SBRs and 190 randomly selected promoter regions that did not show Smad3 binding. Two-way ANOVA was performed to ask whether the fraction of peaks with a SMAD site differed significantly with respect to group (positive vs. negative peaks) and distance from peak midpoint.

To identify enriched transcription factor (TF) binding sites within the SBRs, the set of 190 binding regions identified above was used. The midpoint of each peak was determined, and chromosome sequences consisting of the midpoint nucleotide flanked by 250 bp were examined for the presence of transcription factor binding sites. If necessary, boundaries of regions were adjusted to eliminate overlaps between adjacent sequences. Transcription factor binding sites were identified using the Genomatix software suite [55]. Overrepresented TF binding sites within peaks were identified using the RegionMiner tool. Co-occurrence of TF sites was determined within peak midpoint +/−250 bp regions for SMAD and the six additional most highly overrepresented TF matrices in the 190 binding regions. MatInspector was used to identify TF binding sites in each binding region. Redundant TF sites were removed (for example a generic SMAD site and a SMAD3 site mapped to the same location were counted as one site) and multiple motifs for each TF were collapsed down to a single motif (for example AP1.01 and AP1.02 were reduced to AP1). For each pair of TF factors, the number of peak sequences that contained both sites was determined. *P* values for co-occurrence were calculated using Fisher’s exact test, where TF site co-occurrence in positive peaks were compared to co-occurrence in the set of 190 control promoter regions which showed no Smad3 binding. *P* values were adjusted for multiple comparisons using the Bonferroni correction.

### Global gene expression analysis

In parallel with the Smad3 ChIP-chip analysis, M1 to M4 cells were treated *in vitro* with TGF-β for 1 hour and 6 hours and RNA was isolated for gene expression analysis using RNeasy™ kit (Qiagen). RNA quality was checked on Agilent Bioanalyzer 2100 (Agilent Technologies, Santa Clara, CA, USA). All samples used for microarray analysis have high quality score (RIN >9). A total of 100 ng of RNA was reverse transcribed and amplified using an Ambion WT expression kit following the manufacturer’s instructions. Sense strand cDNA was fragmented and biotinylated using Affymetrix WT Terminal Labeling Kit. Three biological replicates for each condition were hybridized to the Affymetrix GeneChip™ Human ST1.0 in a hybridization oven at 45°C, 60 rpm for 16 hours. Washing and staining were performed on an Affymetrix Fluidics Station 450 using the Affymetrix GeneChip™ Hybridization Wash and Stain Kit containing R-phycoerythrin, strepavidin and biotinylated anti-streptavidin antibody, and Genechips were then scanned on an Affymetrix GeneChip™ scanner 3000 7G. Data was collected using Affymetrix AGCC software. To assess the regulation of target genes by TGF-β in M3 cells *in vivo*, gene expression arrays were also performed for tumor xenografts of M3 cells transduced with pLPCX retrovirus with no insert (M3-CON) or pLPCX expressing a dominant negative type II TGF-β receptor (M3-dnTβRII). Six tumors were arrayed for each genotype group. All expression arrays were normalized by the RMA method using the Affymetrix Expression Console.

### RT-QPCR

RNA prepared by the RNeasy method (Qiagen) from M3 tumors transduced with lentiviruses expressing shGFP (M3-shCON), shSmad3 (M3-shSmad3) or dnTβRII was also analyzed by RT-QPCR. cDNA was synthesized using Superscript III (Invitrogen) and RT-QPCR was performed using Power SBYR Green PCR Master Mix (Applied Biosystems) and an ABI PRISM™ 7900 HT Sequence Detection System (Applied Biosystems). Three biological replicates of each tumor type or each *in vitro* cell treatment condition were used for all RT-QPCR validation experiments. Expression was normalized to PPIA and fold change in expression was calculated relative to the indicated conditions. Primer pairs used for RT-QPCR are given in Additional file 3.

### Integration of ChIP-chip and gene expression array datasets and signature generation

To identify the TGF-β/Smad3-dependent gene expression signature, Smad3 ChIP-chip data and microarray-based gene expression datasets were integrated using Partek Genomic Suite 6.5 (Partek, St. Louis, MO, USA). Gene expression datasets were log2 transformed and quantile normalized. ANOVA analysis was then performed on the set of Smad3 target genes that had been identified by ChIP-chip, to select for TGF-β/Smad3 target genes that were differentially expressed, based on FDR <0.05, across the four target conditions *in vitro* (M3 cells −/+TGF-β and M4 cells −/+TGF-β). Unsupervised hierarchical cluster analysis was performed, in which genes showing the most variation in expression across the four treatment groups as determined above were computed using Euclidean distance metric and average linkage clustering for both ‘genes’ and ‘groups’ and then used to generate heat maps. Smad3 target genes that showed significant regulation by TGF-β treatment *in vitro* were then assessed for regulation *in vivo* by comparing gene expression array datasets generated from M3-CON and M3-dnTβRII tumors (six tumors/genotype group) on the Affymetrix Human 1.0ST array platform. An FDR cutoff of 0.2 was used to identify differentially expressed genes in the *in vivo* datasets. Despite the low stringency cutoff, all 26 genes identified by microarray as being uniquely regulated by TGF-β in M3 cells *in vitro* and also regulated by TGF-β in M3 tumors *in vivo* were validated by RT-QPCR (see Results).

### Meta-analysis of gene expression in clinical breast cancer datasets

Meta-analysis of the association of individual genes or the gene expression signatures with clinical parameters and outcome in human breast cancer array datasets was performed using the GSA tumors function in the online tool GOBO (gene expression-based outcome for breast cancer online) [56,57]. The tumor dataset used for the GOBO analyses consists of a total 1,881 samples, with the following characteristics: 1,225 ER+ tumors, 395 ER- tumors, 927 tumors from patients who received no systemic therapy (untreated) and 326 tamoxifen-treated tumors. All tumors were arrayed on the Affymetrix U133A array and came from 11 independent datasets. Importantly, the GOBO analysis tool allows for directional weighting (positive or negative) of component genes of a signature. Thus genes that were upregulated by TGF-β were assigned a weight of +1, and genes that were downregulated were assigned a weight of −1. Depending on the analysis, we used the directional weightings that we determined *in vitro* or *in vivo,* as indicated in the text. For gene sets such as the TGF-β/Smad3 tumor-suppressor signature (TSTSS), the program computes an averaged gene set expression, including weights, prior to dividing the entire dataset into patient cohorts based on gene expression quantiles. In our analyses, datasets were dichotomized to high (above median) and low (below median) expression values for the gene or gene set in question, and Kaplan-Meier analysis was performed to determine association of gene expression with outcome. Multivariate analyses, and analyses of gene expression patterns across clinical groups were also performed with the GOBO tool.

To assess the performance of the TSTSS in independent breast cancer gene expression datasets obtained using different microarray platforms, the Nederlands Kanker Instituut (NKI) dataset using a custom spotted cDNA array [58], and the Cancer Genome Atlas (TCGA) dataset [59] using a Illumina microarray platform were used (Illumina Inc., San Diego, CA, USA). The NKI dataset has 337 samples (249 ER+ and 88 ER- tumors) and 19 out of the 26 genes of the TSTSS were found in this dataset, while the TCGA dataset has 525 breast tumors (407 ER+ and 118 ER-) and 24 of the 26 TSTSS genes were found. We applied the R package ‘survival’ [54] to estimate the probability of distant metastasis-free survival and overall survival in these datasets using the Kaplan-Meier method. For each sample, we computed the weighted sum of expression of the genes of the TSTSS. From these sums, we defined a factor variable with value High if the sum was greater than the median of the sums, and Low if the sum was not greater than the median. The factor variable and the log rank test were used to test the difference between the two groups. The analysis was applied to all samples, and to ER+ and ER- subgroups. To assess the validity of the signature, permutation analysis was performed for the weight vectors and genes of the TSTSS within the dataset GSE6532. In this dataset, expression data were found for 22 out of the 26 genes. The number of all possible (−1 or 1) binary vectors is 4194304. The Kaplan-Meier analyses of the signatures using all possible weight vectors showed that the *in vivo* TSTSS combination outperformed 97% of all possible combinations. We also tested 10,000 random subsets of the 22 genes with random binary vectors to combine them. The *in vivo* TSTSS combination outperformed 98% of the 10,000 random subset signatures.

### Gene expression indices and correlation analyses

A published metaPCNA index [60] was used as a surrogate for cellular proliferation in the gene expression datasets. The metaPCNA index is the median expression of the top 1% of genes that were most highly positively correlated with the proliferation marker proliferating cell nuclear antigen (PCNA) in the GSE2361 dataset, which represents gene expression profiles from 36 different normal human tissues [61]. We similarly generated a metaEphrin index that consisted of the weighted sum of the top 30 genes most highly positively correlated with EFNA1 in the GSE2361 dataset, using the correlation coefficients as weights. We also generated a metaDiff index, representing luminal differentiation, that is the median expression of a manually curated list of 11 genes that were highly expressed in differentiated luminal or luminal progenitor cells of the normal breast epithelium in multiple studies [62-64]. The genes of the metaDiff index were: FOXA1, CITED1, GATA3, ESR1, PGR, WNT4, KRT19, MUC1, ELF5, KIT, CYP24A1. The Spearman correlation was performed to determine the relationship between the TSTSS and the various indices in individual datasets after the removal of outliers. Outliers were defined as follows: Given a data vector x, let q1 and q3 be the 1st and 3rd quartiles of x, that is, q1 = Q(0.25) the 25% quantile and q3 = Q(0.75) the 75% quantile. Define interquartile range (IQR) = q3-q1. Let m be the median of x. Define the interval [a,b] = [m-1.5*IQR, m + 1.5*IQR]. Any data outside this interval are outliers. By this definition, fewer than six points were dropped from any given dataset. The metaPCNA index was also used as a proliferation surrogate in multivariate analysis of the prognostic power of the TSTSS in the breast cancer datasets.

### Tumorigenesis

For tumorigenesis assays, M3 or M4 cells were suspended in serum-free DMEM/F12 medium, and 5 × 10^5^ cells were injected into the #2 and #7 mammary fat pads of six- to eight-week-old female athymic NCr nu/nu mice. Tumors were measured weekly with calipers and all mice on a given experiment were euthanized with CO_2_ when the first tumor in any experimental group reached 2 cm in diameter. All animal studies were done under a protocol (LC-070) approved by the National Cancer Institute, in accordance with Association for Assessment and Accreditation of Laboratory Animal Care (AAALAC) Guidelines and policies established by the NIH.

### Immunohistochemistry and histopathology

Formalin-fixed, paraffin-embedded tumor xenografts were immunostained with antibodies against cytokeratin 8 (CK8; Troma-1, Hybridoma Bank, Iowa, IA, USA), estrogen receptor α (ER-α; MC-20, sc-542, Santa Cruz Biotechnology Inc.), angiopoietin-like 4 (#18374, Proteintech Group, Chicago, IL, USA) and serpinE1 (AF1786, R&D Systems). Immune complexes were detected using the Vectastain Elite ABC Peroxidase Kit (Vector Labs, Burlingame, CA, USA) and the two-component DAB substrate pack (Biogenex, San Ramon, CA, USA), as directed by the manufacturers. The primary antibody was omitted as a negative control. Images were captured using an Axioplan Universal microscope (Zeiss, Oberkochen, Germany), and immunostaining in five to ten randomly selected high power fields (CK8: 20X objective; ER: 40X objective) was quantitated using ImagePro Plus Software (MediaCybernetics Inc., Silver Spring, MD, USA). To determine the extent of histological differentiation of the tumors, the tumor area occupied by well-differentiated glandular-like structures was assessed by a pathologist as previously described [65].

### Accession numbers

The ChIP-chip and *in vitro* and *in vivo* gene expression microarray data from this publication are available from GEO under the accession number Series GSE34277, consisting of three constituent datasets GSE34270, 34271, 34276.

### Statistics

Statistical analyses of experimental data were done in GraphPad Prism 5.0 (GraphPad Software Inc., San Diego, CA, USA) unless otherwise indicated.

## Results

### Smad3 mediates TGF-β-induced tumor suppression in a model of breast cancer progression

**Figure 1:**
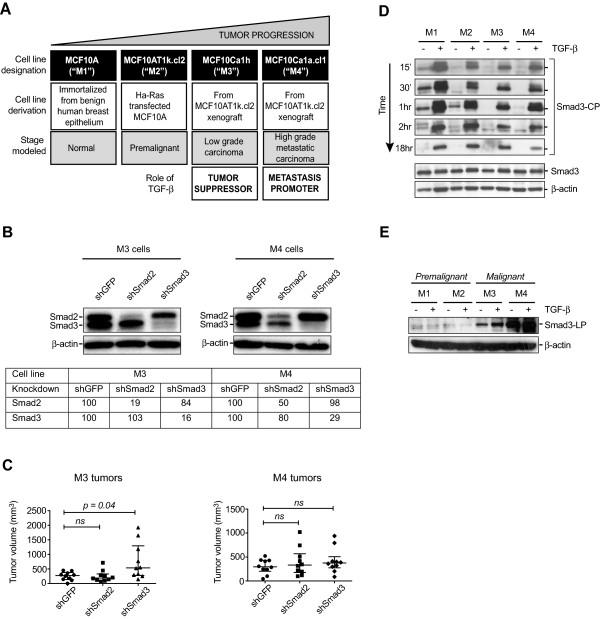
**Smad3 mediates tumor suppression by TGF-β in the MCF10A model of breast cancer progression. (A)** Schematic illustration of the MCF10A-based xenograft model of breast cancer progression. TGF-β has tumor-suppressor activity in M3 cells but this effect is lost in M4 cells and instead TGF-β promotes metastasis. **(B)** Knockdown of Smad2 and Smad3 protein in M3 and M4 cells was verified by Western blot, quantitated relative to the β-actin loading control and normalized relative to the shGFP condition for each cell line. **(C)** Relative contributions of Smad2 and Smad3 to the tumor-suppressive effect of TGF-β. Mice were orthotopically implanted with M3 cells or M4 cells, genetically modified to stably express shSmad2, shSmad3 or the control shGFP, and tumor volumes were assessed after seven weeks (M3) or four weeks (M4). Bars indicate median +/−interquartile range; *P* <0.05 was statistically significant, Mann-Whitney *U* test. ns, not significant. **(D)** Kinetics of Smad3 phosphorylation. Western blot of total Smad3 protein and C-terminal phosphorylated Smad3 (Smad3-CP) levels at various time points after TGF-β treatment in M1 to 4 cells. Total Smad3 is shown for t = 0 h. **(E)** Western blot showing linker phosphorylated Smad3 (Smad3-LP) at 1 hour after treatment with 2 ng/ml TGF-β. TGF-β, transforming growth factor beta.

To address the role of TGF-β at different stages of breast cancer progression, we have used four MCF10A-derived cell lines developed by Miller and co-workers [37,38], as schematized in Figure 1A. Although the parental nontumorigenic MCF10A cell line is ER negative, the derivative cell lines show varying degrees of ER positivity and biological responses to estrogen *in vitro* and *in vivo,* and have other characteristics of ER+ breast cancer (see Methods). Using a dnTβRII to block TGF-β signaling *in vivo*, we previously showed that TGF-β acts as a tumor suppressor in M2 and M3 cells, but not in the closely related M4 cells where TGF-β now acts as a metastasis promoter [25]. Thus in the transition from M3 to M4 the tumor suppressive responses are selectively lost, and the model system provides a valuable platform for the identification of genes specifically involved in TGF-β-driven tumor suppression.

To identify genes at the core of the tumor suppressor program, we decided to focus on direct transcriptional targets of TGF-β, reasoning that hierarchically these would be the most upstream regulators of the program. In the canonical TGF-β signaling pathway, binding of TGF-βs to their cell surface receptors leads to phosphorylation and activation of the signal-transducing components Smad2 and Smad3, which translocate to the nucleus and regulate gene expression. While TGF-β can signal through non-canonical pathways, canonical Smad signaling is thought to be central to TGF-β-driven tumor suppression [66]. Smad2 and Smad3 are highly homologous, but may play non-redundant or even opposing roles in TGF-β signaling [67], so we first wished to determine which Smad was more important for tumor suppression in the MCF10-based breast cancer model. Using shRNA knockdown, we found that Smad3 but not Smad2 mediates the TGF-β-induced tumor suppressive responses in M3 tumors, and this tumor suppressive effect of Smad3 was lost in the more malignant M4 cell line (Figure 1B,C). We then showed that loss of tumor suppression in M4 was not due to changes in Smad3 expression, or duration or extent of Smad3 C-terminal phosphorylation (Figure 1D), which were similar between all four cell lines. Input from other signaling pathways can lead to phosphorylation of Smads on the middle linker region, resulting in loss of select tumor suppressive responses [68]. However, basally high levels of linker phosphorylation of Smad3 were already evident in the malignant M3 cells, which nevertheless still retain their tumor suppressive responses to TGF-β (Figure 1E), so loss of tumor suppression in M4 cells cannot be due to *de novo* Smad3 linker phosphorylation. The level of Smad3 linker phosphorylation in M3 varied somewhat between experiments but was always much greater than in M1 and M2, and was either slightly less than or similar to that in M4. Thus we reasoned that events downstream of Smad3 activation are primarily responsible for the loss of tumor suppression in M4 cells.

### Promoter-wide analysis shows major changes in Smad3 binding patterns with cancer progression

**Figure 2:**
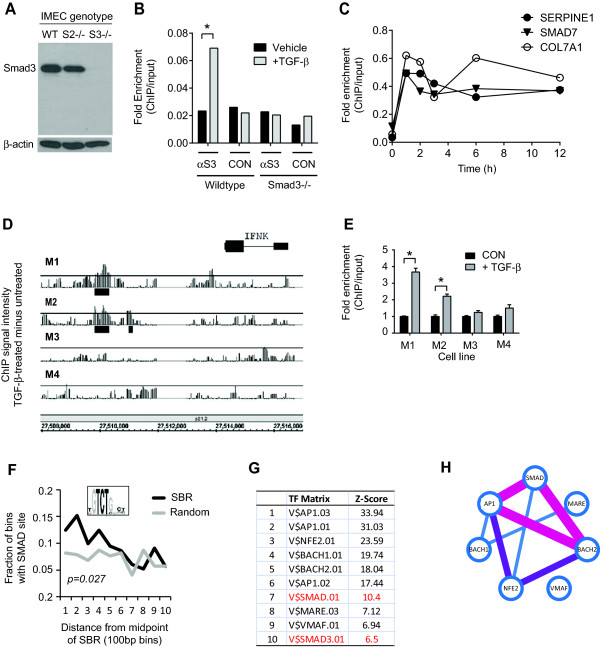
**Identification of Smad3 target genes by ChIP-chip in the MCF10A progression series. (A)** The anti-Smad3 antibody recognizes a unique band in wild type and Smad2 null but not Smad3 null IMECs by Western blot. **(B)** ChIP-QPCR showing ability of Smad3 antibody (αS3) to immunoprecipitate Smad3 bound to the *Smad7* promoter in Smad3 wild-type but not Smad3 knockout mouse embryo fibroblasts. CON, isotype-matched control antibody. **(C)** Time course of Smad3 occupancy at promoters of three previously characterized Smad3 target genes assessed by ChIP-QPCR following treatment of M3 cells with TGF-β. **(D)** Genome browser view (hg18) of Smad3 binding in the promoter of IFNK in M1 to M4 cells. The signal represents the difference between the TGF-β-treated and untreated conditions. The threshold represents signal intensity corresponding to FDR = 0.15. Black rectangles represent regions of significant Smad3 binding. **(E)** ChIP-QPCR validation of Smad3 occupancy at the IFNK locus. Results are mean +/−SD (n = 3) normalized to no TGF-β condition. *P <0.05 for enrichment >2-fold. **(F)** Enrichment of the canonical Smad binding element (SBE) in SBRs. The black line represents 190 high confidence SBRs and the grey line represents 190 random promoter regions with no Smad3 binding. The generic SMAD binding motif is shown. **(G)** Top 10 enriched transcription factor (TF) matrices within +/−250 bp of the center of 190 high confidence SBRs. **(H)** Schematic showing co-occurrence for the most enriched TF motifs. Pairwise analysis of each enriched motif was performed using the Fisher’s exact test with Bonferroni correction. The adjusted *P* values for co-occurrence of pairs of TFs are represented by the connecting lines: *P* <1e-7 (purple), *P* <1e-5 (pink), *P* <1e-2 (black). ChIP, chromatin immunoprecipitation; FDR, false discovery rate; IMEC, immortalized mouse mammary epithelial cells; QPCR, quantitative polymerase chain reaction; SBR, Smad binding region; TGF-β, transforming growth factor beta.

We next hypothesized that loss of tumor-suppressive activity is caused by changes in the spectrum of TGF-β/Smad3 target genes with increasing tumor progression. We therefore performed promoter-wide ChIP-chip analysis using a Smad3-specific antibody (Figure 2A) to identify TGF-β/Smad3 target genes. This antibody immunoprecipitated target DNA from a known Smad3 binding region in the Smad7 promoter in Smad3 wild-type but not Smad3 null mouse mammary epithelial cells (Figure 2B). In the human breast cancer cell lines, we showed that Smad3 binding to known target sites in the promoters of the SMAD7, COL7A1, and SERPINE1 genes in M3 cells was maximal by 1 hour after TGF-β addition, so this time point was selected for the ChIP-chip analysis (Figure 2C). Parallel gene expression studies were performed at both 1 hour and 6 hours after TGF-β addition.

Smad2 and Smad3 can be activated by other TGF-β superfamily members such as the activins [3], as well as by the unrelated kinases Mps1 [69], WNK1 [70] and MPK38 [71], and by advanced glycation end products [72]. In order to focus specifically on TGF-β-driven Smad3 binding, we filtered the ChIP-chip data for loci that showed differential Smad3 occupancy between the untreated and TGF-β-treated states, rather than just analyzing the treated state as has been done previously [73-75]. This strategy yielded 498 TGF-β-induced Smad3 binding regions (SBR) corresponding to 404 annotated genes across all four cells (Additional file 4). Representative results for *IFNK*, a novel gene target that only shows Smad3 binding in M1 and M2 cells, are shown in Figure 2D,E. As expected, the canonical Smad3 binding motif GTCT, or its reverse complement AGAC, were significantly enriched within the SBRs (Figure 2F), though the most enriched transcription factor motifs were those of the AP-1 family (Figure 2G), with Smad motifs frequently co-occurring with AP-1 family motifs in the SBRs (Figure 2H). Enrichment of AP-1 motifs in Smad2/3 binding regions was previously also observed in keratinocytes [73], and probably reflects the ability of Smads to bind directly to the AP1 motif binding site through TGF-β-inducible interactions of Smad3 with c-Fos, c-Jun or Fra1 [76,77].

**Figure 3:**
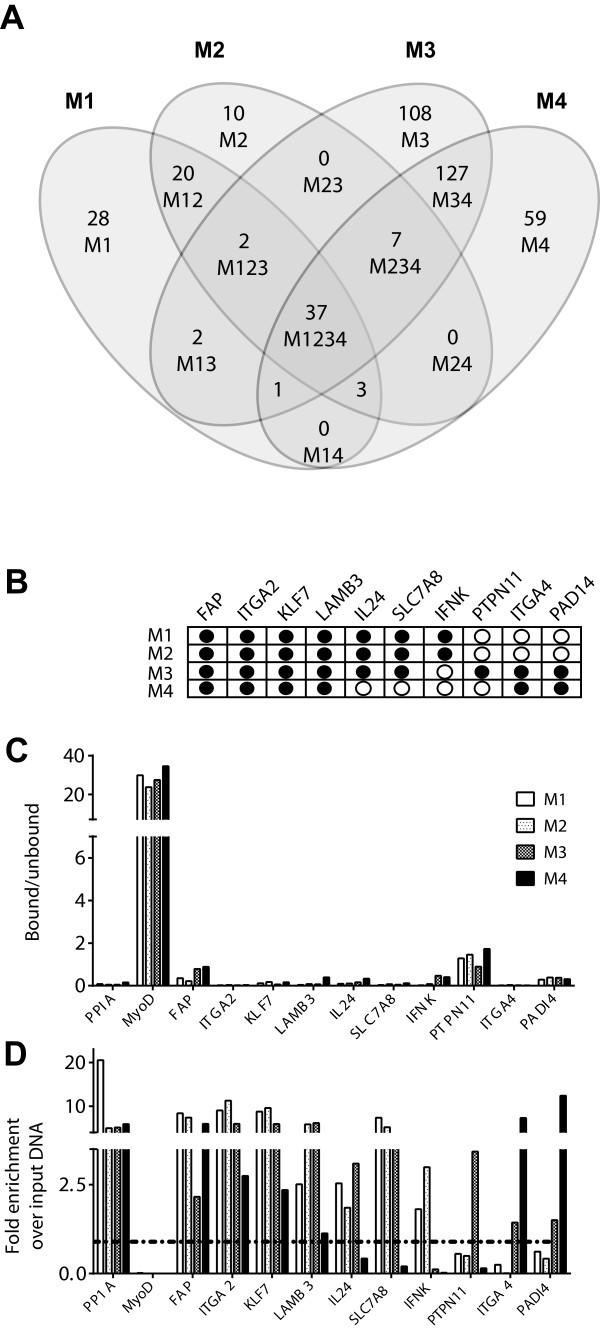
**Smad3 binding differs widely across the progression series. (A)** Petal plot showing the overlap in Smad3 target genes between the different cell lines. The genes involved are given in Supporting Information Table S1 in Additional file 4. **(B)** Representative genes with distinct Smad3 occupancy patterns as confirmed by ChIP-QPCR. Closed circles indicate TGF-β-induced Smad3 occupancy. **(C)** DNA methylation status at promoter regions of each target gene in **(B)** as determined by MeDIP-QPCR. Relative enrichment in the bound (MeDIP) vs. unbound fractions is shown. PPIA and MyoD were controls for unmethylated and highly methylated DNAs respectively. **(D)** QPCR quantitation of target enrichment following ChIP using anti-H3AcK9/14 to identify active chromatin. Enrichment at the SBR was calculated relative to input DNA. PPIA and MyoD were controls for active and inactive promoters respectively. Active chromatin has an enrichment value >1.00 (indicated by threshold line). ChIP, chromatin immunoprecipitation; H3AcK9/14, histone H3 acetylated on lysine 9 or 14; MeDIP, methylated DNA immunoprecipitation; QPCR, quantitative polymerase chain reaction; SBR, Smad binding region; TGF-β, transforming growth factor beta.

Despite the close genetic relatedness of MCF10-derived cell lines, relatively few Smad3 target genes (37/404 = 9.2%) were common to all four lines, with the malignant M3 and M4 cells showing a particularly high proportion of unique targets (Figure 3A and Additional file 4). ChIP-QPCR validation at 25 loci in all four cell lines was performed that broadly confirmed this unexpected finding (Additional file 5). Furthermore, global TGF-β-regulated gene expression showed a similar pattern, with large numbers of unique gene targets in the four cell lines (Additional file 6), confirming that the observation of cell-line dependent gene occupancy and regulation in response to TGF-β/Smad3 is not due to low sensitivity of the ChIP analysis but instead reflects a fundamentally important feature of TGF-β biology. To determine the basis of this phenomenon, we selected 10 target genes representing different patterns of TGF-β-induced Smad3 occupancy across the four cell lines (Figure 3B), and determined whether there were differences between the cell lines in local DNA methylation and chromatin activation state at the SBRs. We found that the target promoters were all hypomethylated in all four cell lines (Figure 3C), so occupancy patterns could not be explained by differential promoter methylation. However, TGF-β only induced Smad3 occupancy at SBRs in regions of chromatin that were activated prior to TGF-β treatment, as assessed by ChIP for the presence of H3AcK9/14 at the SBR in the untreated state (Figure 3D). Thus the spectrum of TGF-β-induced Smad3 binding in the different cell lines reflects preexisting local differences in the activated chromatin landscape. This observation identifies one molecular mechanism that may contribute to the well-known contextuality of TGF-β action.

### Identification of Smad3 target genes that contribute to the tumor suppressive effects of TGF-β

**Figure 4:**
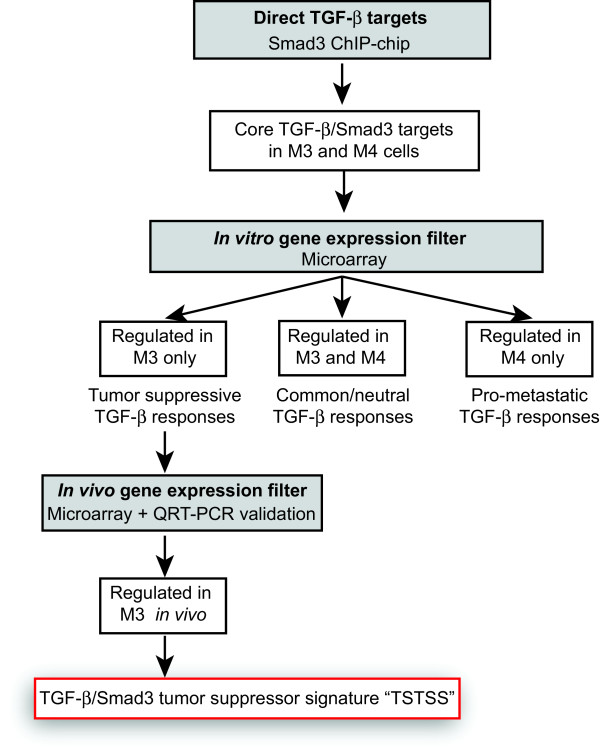
**Strategy for integration of ChIP-chip and gene expression datasets to generate a core TGF-β/Smad3 tumor suppressor signature.** The experimental strategy for identification of the TGF-β/Smad3 tumor suppressor signature (TSTSS) is shown. ChIP, chromatin immunoprecipitation; TGF-β, transforming growth factor beta.

**Figure 5:**
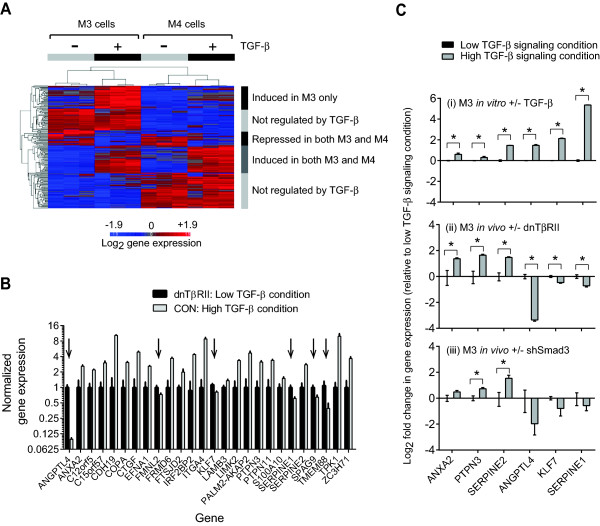
**Generation of core TGF-β/Smad3 tumor suppressor signature. (A)** Unsupervised hierarchical clustering of differentially expressed Smad3 target genes in M3 and M4 cells treated with TGF-β *in vitro* for 6 hours. The 38 genes induced by TGF-β *in vitro* in M3 only were taken forward for further analysis. **(B)** RTQ-PCR validation of the 26 genes out of the original 38 genes that microarray analysis showed to be also regulated by TGF-β in M3 tumors *in vivo*. M3 tumors transduced with the dnTβRII represents the low TGF-β signal condition *in vivo*, while M3 tumors transduced with control lentivirus represents the high TGF-β signal condition *in vivo*. Expression was normalized to the low signal condition for each gene. Results are the mean +/−SEM for three tumors/experimental group. The difference between the high and low TGF-β signaling conditions is statistically significant (*P* <0.05; unpaired *t* test) for all genes shown. Note the six genes, marked by arrows, which are downregulated by TGF-β *in vivo* whereas they were upregulated *in vitro*. **(C)** Smad3 dependence of TGF-β regulation of select target genes *in vivo*. Further RT-QPCR quantitation was performed for six representative genes under the following conditions: (i) M3 cells treated with 5 ng/ml TGF-β (high TGF-β signal condition) or vehicle (low signal condition) *in vitro*; (ii) M3 tumors *in vivo* following transduction with a dnTβRII to block all TGF-β responses (low signal condition) or LacZ control lentivirus (high signal condition); (iii) M3 tumors *in vivo* following transduction with shSmad3 to block Smad3-mediated responses (low signal condition) or shGFP control lentivirus (high signal condition). Results are mean +/−SEM for three to six independent samples/group, normalized to low signaling condition. *statistically significant (*P* <0.05) for high vs. low signaling condition, unpaired *t* test. dnTβRII, dominant-negative type II TGF-β receptor; TGF-β, transforming growth factor beta.

Having identified a set of core TGF-β/Smad3 target genes in the breast cancer model system using the ChIP-chip approach, we next determined which of these were specifically important for the tumor suppressive functions of TGF-β by assessing gene expression. Microarray analysis of gene expression *in vitro* (using a *P* value cutoff of <0.001 for differential expression between the TGF-β-treated and untreated conditions) revealed that approximately 50% of the Smad3 occupied genes in each of the cell lines showed altered gene expression within 6 hours after TGF-β treatment (Additional file 7). Since the tumor-suppressive effect of TGF-β is lost on progression from M3 to M4 cells, we focused our subsequent analysis on these two lines in order to develop a TGF-b/Smad3 tumor suppressor signature (TSTSS), as schematized in Figure 4. First, we compared patterns of TGF-β-regulated expression of Smad3 target genes *in vitro*, reasoning that genes that were regulated in M3 only should be enriched for Smad3-driven tumor-suppressive responses. Unsupervised hierarchical clustering of genes that were heterogeneously expressed across the four conditions (Figure 5A) revealed sets of Smad3 target genes that were (a) uniquely upregulated by TGF-β in M3 (Cluster I: 38 genes total); (b) similarly regulated by TGF-β in M3 and M4 (Clusters III and IV: 65 genes total); and (c) not regulated by TGF-β but basally different between the two cell lines; Clusters II and V). No distinct clusters of Smad3 target genes that were uniquely regulated in M4 cells were identified.

TGF-β effects are highly context dependent, and the *in vivo* microenvironment provides a different set of contextual cues that could affect gene expression. To ask whether the 38 Smad3 target genes that were uniquely upregulated by TGF-β in M3 cells *in vitro* were also regulated by TGF-β *in vivo*, we next analyzed gene expression in M3 tumors with and without TGF-β pathway ablation using a dnTβRII [65]. Microarray analysis of these tumors showed that 26/38 (77%) of the M3 unique targets were also regulated by TGF-β *in vivo,* a finding that was confirmed by RT-QPCR (Figure 5B). Unexpectedly, nearly 25% of these genes were regulated in the opposite direction by TGF-β *in vitro* and *in vivo*, including the hallmark TGF-β response genes *SERPINE1* and *ANGPTL4* (Figure 5B). Since this was a surprising result, we then wished to determine whether the discordant *in vitro*/*in vivo* results reflected an involvement of alternative TGF-β signaling pathways other than Smad3 in regulation of the discrepant genes in the *in vivo* setting. We took representative genes that were concordantly *(ANXA2, PTPN3, SERPINE2)* or discordantly *(ANGPTL4, KLF7, SERPINE1)* regulated between the *in vitro* and *in vivo* conditions and analyzed expression of the same genes in M3 tumors with and without Smad3 knockdown. The results were consistent with a role for Smad3 in the *in vivo* setting as well as *in vitro* (Figure 5C). Thus TGF-β signaling through Smad3 can upregulate or repress the same target genes in a given cell line depending on the local microenvironmental context (*in vitro* vs. *in vivo*). To confirm that the discrepancy in direction of target gene regulation *in vitro* and *in vivo* was not due to major contributions from the stroma in the tumors *in vivo*, we immunostained M3 tumors for ANGPTL4 and SERPINE1 and showed that these proteins were expressed predominantly in the tumor parenchyma (Additional file 8). The data emphasize the critical importance of including an *in vivo* gene expression filter in this type of approach, since clearly the directionality of Smad3 target gene regulation *in vivo* cannot reliably be extrapolated from *in vitro* results.

### TGF-β/Smad3 target genes associated with tumor suppression predict good clinical outcome in human breast cancer datasets

**Figure 6:**
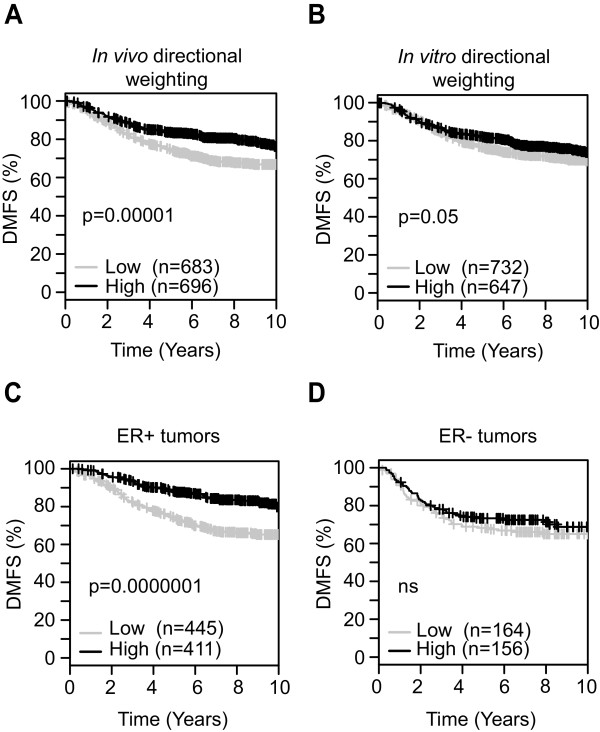
**Meta-analyses correlating TGF-β/Smad3 target genes with outcome in human breast cancer datasets.** Kaplan-Meier analyses were performed using the online GOBO tool to assess the association of the TGF-β-regulated gene sets with distant metastasis-free survival (DMFS) in meta-analyses across multiple breast cancer cohorts (1,379 tumors from eight datasets). Patient datasets were dichotomized to higher than median expression (black) or lower than median expression (grey) of the gene set. *P* values were determined by the log-rank test. **(A,B)** Kaplan-Meier plots for survival of all patients in the GOBO datasets, using the set of TGF-β/Smad3 target genes that were uniquely regulated in M3 (the TSTSS). This gene set was designed to be enriched in genes involved in tumor suppression. Weighting (positive or negative) of individual target genes is based on the directionality of TGF-β-regulated gene expression observed in M3 tumors *in vivo***(A)** or in M3 cells *in vitro***(B)** as indicated. **(C,D)** Correlation of the TSTSS (using the *in vivo* directional weighting) with DMFS in ER+ (n = 856) **(C)**, and ER- (n = 320) **(D)** patient subsets of the GOBO cohorts. ns, not significant. ER, estrogen receptor; GOBO, gene expression-based outcome for breast cancer online; TGF-β, transforming growth factor beta; TSS, transcriptional start site; TSTSS, TGF-β/Smad3 tumor suppressor signature.

Taking the core list of 26 genes that survived the *in vivo* filter, we next asked whether this TSTSS was associated with clinical outcome in human breast cancer datasets. Using the GOBO software [56], which allows a directional weighting (upregulated vs. downregulated) to be assigned to individual genes in a gene set, we performed a meta-analysis of the TSTSS in eight clinical breast cancer gene expression datasets that used the Affymetrix array platform. Twenty out of the 26 genes of the TSTSS were represented in the GOBO clinical datasets (Additional file 9A). High expression of the TSTSS was strongly associated with better distant metastasis-free survival (DMFS) (*P* = 0.00001) in datasets representing 1,379 breast cancers when the *in vivo* directional weighting for expression of the signature genes was used (Figure 6A). Notably, the prognostic power of the signature was greatly decreased (*P* = 0.05) if weighting was used corresponding to the *in vitro* rather than *in vivo* direction of gene regulation (Figure 6B), thus demonstrating the utility of our integrated *in vitro*/*in vivo* approach.

Permutation analysis in the GSE6532 dataset showed that the *in vivo* TSTSS combination outperformed 97% of all possible combinations of the binary weight vectors, and 98% of 10,000 random subsets of TSTSS genes and random binary weight vectors (see Methods for more details). Furthermore, the prognostic power of the *in vitro*/*in vivo* concordant and discordant gene sets when analyzed separately was much lower than that of the full TSTSS (Additional file 10). Using an identical strategy to that used to generate the TSTSS, we derived a ‘generic’ TGF-β signature from the 65 Smad3 target genes that were regulated by TGF-β in both M3 and M4 *in vitro*, of which 24 genes survived the *in vivo* filter (Additional file 9B). As expected, this signature performed much less well than did the TSTSS (Additional file 11), which highlights the importance of enriching the signature for genes functionally associated with tumor suppression in order to make the TGF-β-driven tumor suppressive signal detectable in clinical samples. It should be noted that TGF-β also functions as a tumor suppressor in premalignant M2 cells [25], but the TSTSS is not evident in M2 cells (not shown). We believe this is because TGF-β likely suppresses the premalignant-to-malignant transition (M2) and tumor progression (M3) by different mechanisms. Since the patient datasets represent tumors from later stages in progression, here we focused specifically on the M3 tumor-suppressor signature.

Consistent with our use of an ER+ model to generate the signature and our observations of the strong contextuality of TGF-β-regulated gene expression, the prognostic power of the *in vivo*-weighted TSTSS was restricted to the ER+ tumors only in the GOBO datasets (Figure 6C,D). In multivariate analysis of ER+ patients in the GOBO cohort, lower than median expression of the TSTSS was associated with increased risk of distant metastasis (hazard ratio = 1.85, confidence interval = 1.28 to 2.67; *P* = 0.001; n = 553 evaluable patients), independent of lymph node status, tumor grade, age at diagnosis and tumor size. The signature also prognosticated in three independent cohorts of ER+ breast cancer patients: the NKI cohort using custom spotted cDNA arrays [58], and the TCGA cohort [78] and BT2000/Metabric cohorts [79] both using Illumina arrays (Additional file 12). Thus performance of the signature is robust across different datasets and array platforms. Our data suggest that TGF-β/Smad3-mediated tumor-suppression plays an important role in the natural history of ER+ breast cancer, and that tumor-suppressive effects of TGF-β are still evident in the tumor at the time of surgery and influencing disease outcome for a significant fraction of patients.

### TGF-β/Smad3 effects on tumor cell proliferation and differentiation in breast cancer

**Figure 7:**
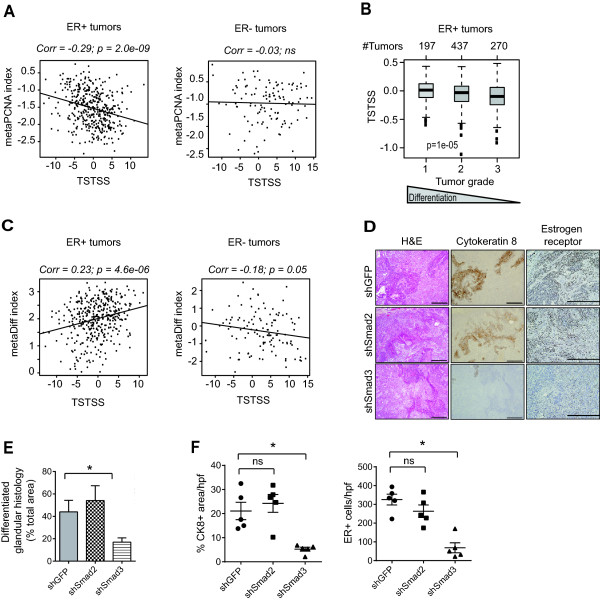
**Loss of TGF-β signaling leads to reduced tumor differentiation. (A)** The TSTSS is weakly anticorrelated with proliferation, as assessed using a metaPCNA index, in ER+ but not ER- breast cancers (TCGA cohort). **(B)** Expression of the TSTSS inversely correlates with tumor grade in ER+ breast cancer (n = 904 patients), as assessed using GOBO tool. **(C)** Correlation between the TSTSS and luminal differentiation, as assessed using a meta-differentiation index, in ER+ and ER- tumors in the TCGA cohort. **(D)** Immunohistochemical staining of cytokeratin 8 (CK8) and ER-α in primary tumors from M3 cells expressing shGFP, shSmad2 or shSmad3. Scale bars represent 100 μm. **(E)** Quantitation of % area occupied by structures with a differentiated glandular histology in M3 tumors expressing shGFP (control), shSmad2 or ShSmad3. Results are mean +/−SEM for five tumors/group. **(F)** Quantitation of CK8 and ER staining was performed using Image-Pro Plus software. Each datapoint represents the mean of five fields/tumor, and results are shown as mean +/−SEM for five tumors/group. *P <0.05 for one-way ANOVA with Dunnett’s multiple comparison test; ns, not significant; hpf, high power field. ER, estrogen receptor; GOBO, gene expression-based outcome for breast cancer online; TGF-β, transforming growth factor beta; TSTSS, TGF-β/Smad3 tumor suppressor signature.

We next asked what biological activities might underlie the tumor-suppressive effects of TGF-β/Smad3 in breast cancer. Inhibition of epithelial cell proliferation is a hallmark activity of TGF-β [3], and we have previously shown that TGF-β strongly inhibits proliferation of M3 cells but not M4 cells *in vitro*[25]. Furthermore, the prognostic power of many breast cancer signatures is driven by proliferation [80]. To address the relationship between the TSTSS and proliferation in the clinical breast cancer datasets, we used a meta-PCNA index as a surrogate for proliferation [60]. There was a weak but highly statistically significant negative correlation between the metaPCNA index and the TSTSS in ER+ breast tumors but not ER- tumors in multiple independent cohorts. Results for the TCGA cohort are shown in Figure 7A. Similar results were obtained for ER+ tumors in the GSE6532/Loi and NKI cohorts (Additional file 13). These results suggest that antiproliferative effects of TGF-β are still active in ER+ tumors. However, the TSTSS still prognosticated independently of proliferation in multiple independent datasets by multivariate analysis (Table 1), suggesting that the tumor-suppressive effects of TGF-β in ER+ breast cancer must also involve additional biological mechanisms.

**Table 1:** Prognostic power of the TSTSS in three independent ER+ breast cancer datasets

		**Breast cancer cohort**								
		**GSE6532 (Loi)**			**NKI**			**TCGA**		
**Variable**	**Ref for HR = 1**	**Hazard ratio**	**CI**	***P *** **value**	**Hazard ratio**	**CI**	***P *** **value**	**Hazard ratio**	**CI**	***P *** **value**
**TSTSS**	**High**	**1.89**	**1.1-3.27**	**0.02211**	**1.81**	**1.12-2.93**	**0.01535**	**2.6**	**1.21-5.58**	**0.0142**
metaPCNA	Low	2.32	1.27-4.24	0.006251	1.67	0.96-2.92	0.07143	0.77	0.38-1.58	0.4788
Age	Low*	1.03	0.63-1.69	0.9	0.52	0.32-0.84	0.007841	1.88	0.94-3.75	0.07393
Node	Negative	0.91	0.54-1.53	0.7113	0.84	0.52-1.34	0.4651	2.33	1.07-5.08	0.03389
Grade	Grade 1	0.99	0.67-1.47	0.9798	1.57	1.1-2.25	0.01365	na	na	na
Size	Continuous	1.33	1.09-1.62	0.005177	1.22	0.95-1.57	0.1109	na	na	na

Multivariate analysis was performed using the Cox proportional hazards model. Nineteen out of the 26 genes of the TSTSS were found in the GSE6532 (Loi) and NKI cohorts, and 24 out of 26 genes were found in the TCGA cohort. The TSTSS was analyzed as a binary variable where high indicates higher than median expression. The hazard ratio refers to distant metastasis-free survival for GSE6532 and NKI, and overall survival for TCGA. The metaPCNA index is a surrogate for proliferation and low indicates lower than median expression. Parameters for the TSTSS are highlighted in bold. *Low is ≤61 yrs (GSE6532); ≤45 yrs (NKI); ≤60 yrs (TCGA). HR, hazard ratio; CI, confidence interval; TSTSS, TGF-β/Smad3 tumor suppressor signature; na, not available.

In ER+ tumors, GOBO meta-analysis showed that the TSTSS was inversely correlated with tumor grade (*P* = 0.00001; Figure 7B), which in part reflects histologic differentiation [81]. A total of 7/26 genes of the TSTSS were annotated for involvement in cellular differentiation (*ANXA2, CTGF, EFNA1, ITGA4, LAMB3, PTPN11, SPAG9*), and we also found that the TSTSS was weakly positively correlated in ER+ but not ER- tumors with a meta-differentiation index (see Methods for definition) that reflects luminal differentiation (Figure 7C). To demonstrate a causal role for TGF-β/Smad3 in regulating breast cancer differentiation, we showed that knockdown of Smad3, but not Smad2, was associated with reduced development of well-differentiated glandular-like structures in M3 tumors (Figure 7D,E), and a significant reduction in expression of the differentiated luminal markers cytokeratin 8 (CK8) and ER (Figure 7D,F). Thus the tumor-suppressive effects of TGF-β in ER+ breast cancer include a role in enhancing cellular differentiation, and Smad3 is a critical mediator of this activity.

### Ephrin signaling contributes to tumor-suppressive effects of TGF-β in ER+ breast cancer

**Figure 8:**
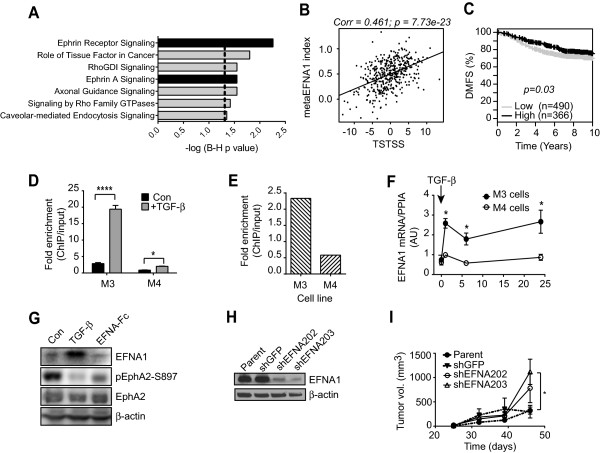
**Ephrin signaling contributes to the tumor suppressive effects of TGF-β in ER+ breast cancer. (A)** Pathway enrichment in the TSTSS assessed by Ingenuity Pathway Analysis. Fisher’s exact test with Benjamini-Hochberg (B-H) correction. The dotted line represents the *P* <0.05 significance threshold. **(B)** Correlation of TSTSS with meta-EFNA index in ER+ tumors of the TCGA cohort. **(C)** Association of meta-EFNA index with distant metastasis-free survival (DMFS) in ER+ breast cancer (n = 856 patients) using the GOBO tool. **(D)** Smad3 ChIP-QPCR at the EFNA1 locus in M3 and M4 cells. **(E)** ChIP-QPCR for H3AcK9/14 at the EFNA1 locus to identify active chromatin. **(F)** Time course of EFNA1 mRNA induction by TGF-β (2 ng/ml) in M3 and M4 cells. Results are mean +/−SEM of three determinations. *P <0.05. **(G)** Western blot of effect of TGF-β treatment of M3 cells on Ephrin A1 (EFNA1) expression and oncogenic signaling through phosphorylation of the EphA2 receptor on S897. EFNA-Fc was used as a positive control for activation of the EphA2 signaling path. **(H)** Western blot showing knockdown of EFNA1 in M3 cells. **(I)** Knockdown of EFNA enhances tumorigenesis in M3 cells. n = 8 to 10 mice/group. *P >0.05 one-way ANOVA, Tukey’s multiple comparison test. ChIP, chromatin immunoprecipitation; ER, estrogen receptor; GOBO, Gene expression-based Outcome for Breast cancer Online; H3AcK9/14, histone H3 acetylated on lysine 9 or 14; QPCR, quantitative polymerase chain reaction; TGF-β, transforming growth factor beta; TSTSS, TGF-β/Smad3 tumor suppressor signature.

To begin to explore molecular mechanisms underlying TGF-β-driven tumor suppression, we performed Ingenuity Pathway Analysis on the signature genes. Network analysis was relatively uninformative for this small number of genes and did not provide any useful leads other than indicating that a number of the signature genes were subject to regulation by ubiquitination (Additional file 14). However, pathway analysis identified ephrin receptor signaling as the most enriched pathway in the TSTSS (Figure 8A), with the genes involved being EFNA1, ITGA4, LIMK2 and PTPN11. The ephrin ligands and ephrin receptors are a large family of membrane-bound proteins that signal bidirectionally in a cell-contact-dependent manner. Ephrin-A1 (EFNA1) ligand binding to the EphA2 receptor at sites of cell-cell contact maintains epithelial phenotype and integrity in part by downregulating Akt, Rho/Rac and Ras pathway signaling [82-84]. In contrast, unligated EphA2 becomes phosphorylated by Akt on Ser897. This phosphorylation event results in loss of suppressive effects on the Ras/Erk pathway and enhanced pro-oncogenic signaling through Akt and Rac1, ultimately leading to increased cell migration, invasion, proliferation and survival [84]. Thus depending on the balance of ligand and receptor, ephrin pathway signaling can be associated with pro-oncogenic or anti-oncogenic outcomes.

To investigate the interrelationship between ephrin signaling and TGF-β, we generated a metaEphrin index consisting of the top 30 genes most highly correlated with EFNA1 mRNA in normal human tissues, and we showed that this index was strongly positively correlated with the TSTSS in ER+ breast cancer datasets (see Figure 8B for the TCGA cohort; similar results were also seen for the GSE6532 and NKI cohorts in Additional file 13). The index was also weakly associated with good outcome in ER+ breast cancers by GOBO meta-analysis (Figure 8C), suggesting that ephrin pathway signaling might contribute to the tumor-suppressive effects of TGF-β in ER+ breast cancer. Using ChIP-QPCR, we confirmed that TGF-β induced Smad3 occupancy at the *EFNA1* promoter to a much greater extent in M3 than M4 cells (Figure 8D), reflecting a more active state of the chromatin at the *EFNA1* promoter in M3 than M4 cells under basal conditions (Figure 8E). As expected, *EFNA1* mRNA expression was upregulated by TGF-β treatment in M3 and not M4 cells (Figure 8F). Upregulation of Ephrin-A1 protein by TGF-β in M3 cells *in vitro* was confirmed by Western blot and was associated with a corresponding decrease in pro-oncogenic phosphorylation of the receptor EphA2 on Ser897 (Figure 8G). Thus by upregulating Ephrin-A1, TGF-β treatment can repress pro-oncogenic signaling through the ephrin pathway in M3 cells. To demonstrate a functional role for Ephrin-A1 in tumor suppression, we used shRNA knockdown in M3 cells (Figure 8H) and showed that ephrin-A1 knockdown significantly increased primary tumorigenesis (Figure 8I). Thus activation of Ephrin-A1 signaling by TGF-β may contribute to the tumor-suppressive effects in ER+ breast cancer.

## Discussion

The highly pleiotropic nature of the TGF-β signaling pathway creates significant hurdles that must be overcome before the complex roles of this pathway in tumorigenesis can be understood and therapeutically exploited in breast cancer. Our integrated genomic approach is the first that specifically isolates the tumor-suppressive gene expression responses to TGF-β from the tumor-promoting or tumor-unrelated responses, and our results suggest that TGF-β-driven tumor-suppressive responses contribute importantly to good clinical outcome in a subset of patients with ER+ breast cancer. A number of interesting features of TGF-β tumor biology were uncovered in this study and the broader implications are discussed further below.

Previous genome-wide ChIP studies have shown considerable differences in Smad target genes between cell lines of different origins [73,74,85]. However, here we found that the spectrum of Smad3 target genes differed to a surprising extent between four highly related breast-derived cell lines, the parental MCF10A line (M1) and its three premalignant or malignant derivatives (M2, M3, M4), despite their common origin and identical test conditions. Furthermore, we showed that TGF-β-induced Smad3 binding occurred in regions of chromatin that were already activated prior to TGF-β treatment, suggesting that the spectrum of Smad3 binding is highly sensitive to preexisting local differences in the epigenetic landscape. This feature of Smad3 effector activity may contribute significantly to the known contextuality of TGF-β action, and raises the possibility that TGF-β-induced Smad3 promoter occupancy mostly serves to fine-tune ongoing transcriptional programs in this model rather than initiating new programs. Consistent with this hypothesis, it was recently shown that during embryonic development Smad3 binds primarily to promoter regions that are already occupied by the master transcription factors for the developmental stage or lineage [86].

Our novel observation that the direction of regulation of Smad3 target genes by TGF-β can differ *in vitro* and *in vivo* identifies an additional new facet of TGF-β contextuality that is highlighted by the effects of TGF-β on *ANGPTL4. ANGPTL4* was previously identified as a metastasis-promoting gene that was upregulated by TGF-β in MDA-MB-231 cells, an ER-negative breast cancer model in which TGF-β has lost its tumor-suppressive activity and instead promotes progression [34]. While *ANGPTL4* was upregulated by TGF-β in both MDA-MB-231 and M3 cells *in vitro,* we found that *ANGPTL4* was actually downregulated by TGF-β/Smad3 *in vivo* in the M3 tumors where TGF-β functions as a tumor suppressor. This is in contrast to the upregulation *in vivo* of *ANGPTL4* in MDA-MB-231 tumors where TGF-β functions as a pro-progression factor. Thus the *in vitro* condition serves to identify TGF-β target genes, but it does not indicate the direction of their regulation in the *in vivo* context and hence cannot predict the critical issue of biological outcome (tumor suppression vs. tumor progression). Identifying the factor that causes the direction of expression of certain Smad3 target genes to flip *in vivo* will be an interesting challenge for the future.

Our integrated genomic strategy allowed us to dissect out a core TGF-β/Smad3 gene signature that specifically reflected the tumor-suppressive activities of TGF-β *in vivo* and was associated with good outcome in multiple independent ER+ breast cancer cohorts. In multivariate analysis, below-median expression of the signature was associated with a two-fold increased hazard ratio for development of distant metastases. This finding strongly suggests that the tumor suppressive effects of TGF-β persist and limit progression in a significant fraction of breast cancers at the time of clinical intervention. Importantly, our TGF-β/Smad3 signature only prognosticated well when the signature genes were weighted for the direction of regulation that was seen *in vivo* and not *in vitro*. Thus two key features of our approach were critical for identifying a discernable tumor suppressor signal for TGF-β in the clinical datasets. One was the use of closely related breast cancer cell lines with and without an intact tumor-suppressive response so that TGF-β-regulated genes that were specifically involved in tumor suppression could be readily identified, and the other was the coupling of the *in vitro* discovery steps with *in vivo* validation. Prognostic TGF-β signatures have previously been generated through strategies that did not explicitly separate the different activities of TGF-β [11,32,34,36]. Almost universally, the signatures are associated with poor outcome in breast cancer patients, suggesting that they primarily capture the pro-oncogenic effects of TGF-β [18,32,34,36]. In the one exception, a TGF-β signature reflecting basal gene expression differences between wild-type and TGFBR2 knockout mouse mammary tumor cells weakly correlated with good outcome in ER+ breast cancers [11]. However, there is little overlap between this signature and our own, suggesting that the different approaches used are capturing complementary aspects of the underlying TGF-β biology. Thus the TGFBR2-based signature implicates TGF-β in the suppression of local inflammation in the tumor microenvironment [11], whereas ours highlights tumor cell-autonomous effects of TGF-β on tumor cell proliferation and differentiation (see later).

We found that high expression of our TGF-β/Smad3 tumor-suppressor signature was associated with good outcome only in patients with ER+ tumors, suggesting that TGF-β plays a particularly important role in limiting progression of this breast cancer subtype. Consistent with these results, TGF-β1 was previously identified as a differentially expressed hub in gene expression networks derived from normal luminal ER+ breast epithelial cells, but not from ER- cells [87], and TGF-β was shown to restrain the proliferation of ER+ mammary cells in normal mice [88]. Interestingly however, we found higher overall expression of the signature genes in ER- breast cancer (Additional file 15), although no association with clinical outcome was observed in this tumor subtype. This observation suggests that that the TGF-β/Smad3 tumor-suppressor program, while still detectable, has been functionally overridden or subverted by the oncogenic pathways that are activated in ER- tumors. It is currently not clear whether TGF-β has distinct tumor-suppressive effects in ER- tumors that are not captured by our signature, since our signature was developed using an ER+ model. However, given our demonstration of the strong context dependence of Smad3 binding and thus of TGF-β effects, we believe that there may not be a universal signature for TGF-β-driven tumor suppression. Thus the very important goal of developing gene signature-based predictive biomarkers for patient inclusion/exclusion from clinical trials with TGF-β antagonists is a challenging one, and our data suggest that such signatures will likely have to be tailored to the specific tumor subtype. Based on our current findings, we propose that patients with ER+ tumors and high signature expression would not be good candidates for TGF-β antagonist therapy, but that the mere presence of this particular TGF-β tumor-suppressor signature in ER- breast cancer patients would not necessarily be a contraindication for such therapy.

Further analysis of our signature gave insights into the mechanisms of tumor suppression by TGF-β in breast cancer. High expression of the signature was inversely correlated with proliferation index and tumor grade, suggesting that the known antiproliferative and differentiation-promoting effects of TGF-β do contribute to tumor suppression in human breast cancer. We had previously shown that TGF-β can induce differentiation in this breast cancer model [65], and here we demonstrated that this effect is mediated by Smad3. However, since the signature still prognosticated independently of proliferation and tumor grade in multivariate analysis, there are likely to be additional as yet unidentified biological activities that also contribute to the TGF-β-driven tumor suppression. In terms of molecular mechanism, our signature implicated Ephrin-A1 as a novel downstream mediator contributing to TGF-β-driven tumor suppression. Like TGF-β signaling, ephrin signaling can have pro-oncogenic or anti-oncogenic effects, depending on the relative levels of ephrin ligands and receptors, and the nature of the target cell [82-84]. In breast cancer model systems, Ephrin-A1 signaling through the EphA2 receptor on the tumor cell can inhibit tumorigenesis [89,90], whereas excess unliganded EphA2 promotes tumorigenesis through enhanced proliferation and migration [90,91]. However, tumor-derived Ephrin-A1 ligand can also have pro-oncogenic effects by promoting tumor angiogenesis through the stimulation of EphA2 signaling on endothelial cells [92]. Thus, as for TGF-β, a complex balance of biological activities is at play. Our bioinformatic data revealed a strong statistical relationship between TGF-β tumor suppression and ephrin signaling in ER+ breast cancer datasets, and we demonstrated experimentally that tumor-autonomous ephrin signaling suppresses tumorigenesis in the M3 breast cancer model. Thus enhanced ephrin signaling plausibly contributes to tumor suppression by TGF-β in ER+ breast cancer.

## Conclusions

We have generated a TGF-β/Smad3-driven gene expression signature that specifically captures the tumor-suppressive effects of TGF-β in ER+ breast cancer. High expression of this signature was associated with good clinical outcome in multiple ER+ breast cancer cohorts, suggesting that tumor-suppressive effects of TGF-β are still active and slowing disease progression at the time of surgery in a significant fraction of breast cancer patients. Clearly such patients should be excluded from treatment with therapeutic TGF-β antagonists. At a molecular level of resolution, we found that cellular responses to TGF-β are even more sensitive to contextual cues than was previously appreciated, which suggests that distinct TGF-β signatures may have to be generated for different tumor types or subtypes. However, using integrated *in vitro*/*in vivo* strategies such as ours, it is clearly possible to assess whether the good or the bad sides of TGF-β dominate in determining disease outcome. Such information will set the stage for safer and more effective therapeutic exploitation of this important signaling pathway in cancer.

## Abbreviations

bp: base pair; ChIP: chromatin immunoprecipitation; CK8: cytokeratin 8; DMEM: Dulbecco’s modified Eagle’s medium; DMFS: distant metastasis-free survival; dnTβRII: dominant-negative type II TGF-β receptor; ER: estrogen receptor; FDR: false discovery rate; GOBO: gene expression-based outcome for breast cancer online; H3AcK9/14: histone H3 acetylated on lysine 9 or 14; IMEC: immortalized mouse mammary epithelial cells; M1: MCF10A cell line; M2: MCF10AT1k.cl2 cell line; M3: MCF10Ca1h cell line; M4: MCF10Ca1h.cl1 cell line; MeDIP: methylated DNA immunoprecipitation; PBS: phosphate-buffered saline; PCNA: proliferating cell nuclear antigen; QPCR: quantitative polymerase chain reaction; SBE: Smad binding element; SBR: Smad binding region; shRNA: short hairpin RNA; TF: transcription factor; TGF-β: transforming growth factor beta; TSS: transcriptional start site; TSTSS: TGF-β/Smad3 tumor-suppressor signature.

## Competing interests

The authors declare that they have no competing interests.

## Authors’ contributions

MSa, MK, BT, YY, MPL and LMW participated in the conception and design of the experiments. MSa, MK, BT, YY and YN performed the experiments, and KCF, MSh, JW and MAW provided technical assistance and expertise in molecular analyses and tumorigenesis studies. AMM, HHY, RJC and MPL all contributed critically to design and performance of the statistical and bioinformatic analyses. LMW and MSa wrote the manuscript with suggestions from all authors. All authors read and approved the final manuscript.

## Supplementary Material

Additional file 1 **Immunostaining for estrogen receptor in tumors from M3 and M4 cells.** Xenografted tumors from M3 and M4 cells were immunostained for estrogen receptor alpha (ERα) as described in Methods. Extensive ERα staining (brown nuclei) is apparent in well-differentiated regions of the M3 tumors, but is also seen in regions of the more poorly differentiated M4 tumors. H&E, hemotoxylin and eosin. Click here for file

Additional file 2 **Primer pairs for QPCR.** All primers are in 5′ to 3′ orientation. Click here for file

Additional file 3 **Primer pairs for RT-QPCR.** All primers are in 5′ to 3′ orientation. Click here for file

Additional file 4 **Location of Smad3 binding regions and their TGF-β-induced occupancy by Smad3 in the four cell lines.** Location of the 404 annotated SBRs and their occupancy in M1 to M4 cells was determined from the ChIP-chip analysis. +, occupied; −, unoccupied. Genome coordinates are Hg18. Click here for file

Additional file 5 **ChIP-QPCR validation of Smad3 target genes with different occupancy patterns between the four cell lines. (A)** A total of 25 Smad3 target genes identified by TAS analysis of ChIP-chip data using an FDR of 0.15 were validated by ChIP-QPCR across all four cell lines and the pattern of gene occupancy was compared between the two methods. The table gives the summary of the results. ChIP-QPCR peaks were scored positive if TGF-β-induced occupancy was significant (*P* <0.05), and ≥2-fold over untreated. A total of 65/67 of the Smad3 binding regions identified by TAS from ChIP-chip in one or more of the cell lines were validated by QPCR. However, QPCR was more sensitive and identified an additional 11/33 instances of Smad3 binding in genomic regions that were called negative in one or more of the cell lines by TAS (see for example Smad3 occupancy of *LAMB3* promoter in M1 and M2). Thus the experimentally determined FDR was 14%, with the majority (85%) of the false calls by ChIP-chip being false negatives. IL31RA is included as an example of a gene that did not show Smad3 occupancy in the ChIP-chip analysis. **(B)** Representative ChIP-QPCR validation results are given for genes that show different patterns of Smad3 promoter occupancy between the four cell lines by ChIP-chip. Results are mean +/−SEM for three replicates. *Smad3 occupancy was induced ≥2-fold by TGF-β and was statistically significant (*P* <0.05; unpaired *t* test). *IL31RA* was selected as a gene that did not show Smad3 binding by ChIP-chip. αS3, anti-Smad3 antibody; CON, control IgG. Click here for file

Additional file 6 **Petal plot showing patterns of TGF-β-regulated gene expression in M1 to M4 cells.** Global TGF-β-regulated gene expression was determined by microarray analysis at the 6 hour time point for all four cell lines. Using a fold-change cutoff of 1.5x and a significance cutoff of *P* = 0.001, a total of 563 genes were found to be significantly changed in their expression across the four cell lines. The majority of these genes were unique to the individual cell lines. Click here for file

Additional file 7 **Expression of Smad3 occupied genes *in vitro.*** Considering only those genes that showed TGF-β-induced Smad3 occupancy, the fraction of TGF-β/Smad3 target genes showing regulated mRNA expression at 1 hour or 6 hours was determined from the microarray analysis using a *P* value cutoff of <0.001 for differential expression between the TGF-β-treated and untreated condition for a given cell line and time point. SBR, Smad3 binding region. Click here for file

Additional file 8 **Immunostaining of M3 tumors for ANGPTL4 and SERPINE1.** M3 tumors were immunostained for ANGPTL4 and SERPINE1 as described in Methods. Immunostaining for both proteins was observed predominantly in the tumor parenchyma (T) and not in the stroma (S). Scale bar represents 25 μm. Click here for file

Additional file 9 **Smad3 target genes uniquely regulated by TGF-β in M3 cells, and Smad3 target genes commonly regulated by TGF-β in both M3 and M4 cells.** From the gene expression microarray data, 38 TGF-β/Smad3 target genes were found to be uniquely regulated by TGF-β in M3 and not in M4 cells *in vitro* (Tab A). A total of 65 TGF-β/Smad3 target genes were found to be regulated by TGF-β in both M3 and M4 cells *in vitro* (Tab B). The tables summarize the direction of regulation of these genes by TGF-β in *in vitro* and *in vivo*. The direction of regulation *in vivo* was determined by comparison of gene expression array data from M3 tumors with or without overexpression of a dnTβRII. The table also indicates which of the genes that survived the *in vivo* filter were represented in the GOBO clinical breast cancer array datasets. The 26 genes that were uniquely upregulated by TGF-β in M3 cells *in vitro* and *in vivo* were validated by RT-QPCR of the tumors (Figure 5). Key: 1 = upregulated by TGF-β; 0 = not regulated by TGF-β; −1 = downregulated by TGF-β; NA, not applicable. For clarity, the final weighted TSTSS signature is also given in Tab C. *The table is given as an Excel Spreadsheet with three tabs: Tab A, Tab B and Tab C*. Click here for file

Additional file 10 **Prognostic power of the *in vitro*/*in vivo* concordant and discordant genes sets from the TSTSS when analyzed separately.** Kaplan-Meier survival curves and multivariate analyses were generated within the GOBO breast cancer datasets using **(A)** only genes whose direction of regulation by TGF-β was concordant between *in vitro* and *in vivo*; **(B)** only genes whose direction of regulation was discordant *in vitro* and *in vivo*; and **(C)** the full TSTSS which includes both gene sets. Of the 26 genes of the TSTSS, 20 were concordant and 6 were discordant. A total of 16 of the 20 concordant genes were found in the GOBO datasets (*not found: ANXA2, C15orf57, FRMD6 and IRF2BP2*). Four of the six discordant genes were found in the GOBO datasets (*not found: FMNL2 and TMEM88*). Click here for file

Additional file 11 **Performance of a ‘generic’ TGF-β signature in breast cancer cohorts using the GOBO meta-analysis tool.** Kaplan-Meier analyses were performed using the online GOBO tool to assess the association of the TGF-β-regulated gene sets with distant metastasis-free survival (DMFS) in a meta-analysis across the multiple breast cancer cohorts of the GOBO dataset (1,379 tumors from eight cohorts). Patient datasets representing all tumors were dichotomized to higher than median expression (black) or lower than median expression (grey) of the gene set. *P* values were determined by the log-rank test. This figure shows Kaplan-Meier plots for survival of all patients in the GOBO datasets, using a ‘generic’ TGF-β signature derived from the set of TGF-β/Smad3 target genes that were regulated in both M3 and M4 cells, and thus not enriched for tumor-suppressor activity. Weighting (positive or negative) of individual target genes is based on the directionality of TGF-β-regulated expression of this gene set in M3 cells *in vitro***(A)** or in M3 tumors *in vivo***(B)** as indicated. The list of genes involved is given in Additional file 9. Note the greatly reduced statistical power of this signature when compared to the use of the TSTSS tumor-suppressor signature in the same cohorts (Figure 6A). Note also that high expression of the generic TGF-β signature is associated with either good or bad outcome depending on whether the *in vitro* or *in vivo* directional weighting was used, again illustrating the strong influence that the biological context in which the signature was generated has on its subsequent performance in the clinical datasets. Click here for file

Additional file 12 **Performance of the TSTSS in independent breast cancer cohorts.** Kaplan-Meier analyses were performed using the R Package ‘Survival’ to assess the association of the TGF-β-regulated gene sets with distant metastasis-free survival (DMFS) or overall survival (OS) in three independent breast cancer datasets that were not included in the GOBO meta-analysis as they used different gene expression array platforms. Patient datasets were dichotomized to higher than median expression (black) or lower than median expression (grey) of the gene set. *P* values were determined by the log-rank test. Performance of the TSTSS (*in vivo* weighting) is shown for ER+ breast cancer datasets from **(A)** the Nederlands Kanker Instituut (NKI: n = 249), **(B)** the Cancer Genome Atlas (TCGA) cohort (n = 407) and **(C)** the BT2000/Metabric (n = 1508) cohorts. (D) GSE6532 (Loi) is a component dataset from the GOBO cohorts using the Affymetrix array platform that was reanalyzed using the same R Package method for direct comparison. Click here for file

Additional file 13 **Correlation between the metaPCNA index or the metaEphrin index and the TSTSS in additional ER+ breast cancer cohorts.** The metaPCNA index **(A)** is a surrogate for proliferation and the metaEphrin index **(B)** is a surrogate for ephrin pathway activation in normal cells. More details on the indices are given in Methods. The GSE6532 (Loi) dataset contains 262 ER+ tumors, and the Nederlands Kanker Instituut (NKI) dataset contains 249 ER+ tumors. The Spearman correlation coefficient is given. Click here for file

Additional file 14 **Network analysis on the genes of the TSTSS.** Ingenuity Pathway Analysis was performed to identify networks formed by the 26 genes of the TSTSS, and the top two networks are shown. Network 1 (score 32) is associated with the following network functions: Cardiovascular System Development and Function, Embryonic Development and Function. Network 2 (score 29) is associated with Cell Cycle, Digestive System Development and Function, and Cancer. Red indicates TGF-β/Smad3 target genes that were upregulated by TGF-β *in vivo*, and green indicates downregulated target genes. White shows non-target genes that were used to generate the networks. Click here for file

Additional file 15 **Relative expression of the TSTSS in different human breast cancer subtypes.** Analyses were done using the GOBO algorithm applied to all breast cancers in the GOBO database. **(A)** TSTSS expression in breast cancers stratified by ER status. **(B)** TSTSS expression in breast cancers stratified by intrinsic molecular subtype. HER2, HER2 amplified; Lum, luminal. The numbers of tumors in each category is given at the top of the figure. ANOVA *P* values. Click here for file
