# Supplementary material for: An integrated genomic approach identifies persistent tumor suppressive effects of transforming growth factor-β in human breast cancer
Source: Breast Cancer Res. 2014 Jun 2;16(3):R57. doi: 10.1186/bcr3668 (PMC4095608; doi:10.1186/bcr3668)
Supplement: Additional file 1 — Immunostaining for estrogen receptor in tumors from M3 and M4 cells. Xenografted tumors from M3 and M4 cells were immunostained for estrogen receptor alpha (ERα) as described in Methods. Extensive ERα staining (brown nuclei) is apparent in well-differentiated regions of the M3 tumors, but is also seen in regions of the more poorly differentiated M4 tumors. H&E, hemotoxylin and eosin. [file bcr3668-S1.docx]

**Additional file 1: Immunostaining for estrogen receptor in tumors from M3 and M4 cells** Xenografted tumors from M3 and M4 cells were immunostained for estrogen receptor-a (ERα) as described in Methods. Extensive ERα staining (brown nuclei) is apparent in well-differentiated regions of the M3 tumors, but is also seen in regions of the more poorly differentiated M4 tumors. H&E, hemotoxylin and eosin.
