# Supplementary material for: An integrated genomic approach identifies persistent tumor suppressive effects of transforming growth factor-β in human breast cancer
Source: Breast Cancer Res. 2014 Jun 2;16(3):R57. doi: 10.1186/bcr3668 (PMC4095608; doi:10.1186/bcr3668)
Supplement: Additional file 2 — Primer pairs for QPCR. All primers are in 5′ to 3′ orientation. [file bcr3668-S2.docx]

**Additional file 2: Primer pairs for Q-PCR** All primers are in 5’ to 3’ orientation

| **TARGET** | **FORWARD PRIMER** | **REVERSE PRIMER** |
| --- | --- | --- |
| ***Mouse*** | | |
| mSmad7_SBR | TTT TAA AGC GAC AGG GTG TCT AGA | TCT GCT CGG CTG GTT CCA |
| mPpia | TCG TGC CGC TTT GCA GA | TGT CGA AGA ACA CGG TGG G |
| ***Human*** | | |
| MYOD | AGC GTT TCC CGC GGA TA | CGG CAC GCC CTT TCC A |
| PPIA | CCA GGC TCG TGC CGT TT | GAC CAT GGC TAA TAG TAC ACG GTT T |
| ABLIM3_SBR | TGG CCC CTG GAC ACG TT | CCT TTG GCC TTC GAG AGC TT |
| ADRA1B_SBR | TGC TCC TCC CCT TCC TTT G | TGG TCA AGT CCA TGG GTC AA |
| ANXA2_SBR | GTG CTA CTG TAG GTT ACA AAC ACT GGT A | TGC AGG CTC AGC GTA TGC T |
| CLSTN1_SBR | GGA ATG GAC TTG ATC CTA CAG TCA | CCT TCC CCA GGT CAC ATC AC |
| COL7_SBR | GCT TCT TGC CTG TCA AGG AGA TC | ACT CCT GTG ATT CAG CCA TCT GT |
| EEF1E1_SBR | CAA GCA TCC AAG AGA TCA TGA AGT | TGA TAG TCA TTG CTT GGG AAG AGA |
| F3_SBR | CAT GAC CCA AAG CAC ACT TCA | TGG GAG AGT TCC TAA GTG GAG TAT AAA |
| FAP_SBR | TGA ACT GTT TCT GTG TAA GTC CTC TCA | CAG GCT AGG GCA GAA CTT CTG A |
| GAB1_SBR | GCA CCG GAA GCA AAC ATT TAT T | AAG GCT CAG CCA TCT CAA TGA |
| IFNK1_SBR | TGA GTA ATA TGG ATG ATC AAT GTG TTA CTC | TTG GCA TCT ACT GAT AAG ATT TAT TGC |
| IL24_SBR | GGG TGT TGG TGC CAG AGA GA | GAG ATG AAA GAA AAG AGG AGA GTG AGT A |
| IL31RA_SBR | TTT CCT TCA TCT CCT TCA TTG TCT AA | GGT AAT AGC TGA AAC AGT GAG AGT ATC TTT |
| ITGA2_SBR | AAA TGC CTT CAG GTG TTT GTC A | AAA TTA TAT AGT CCC AGG CTC TTT CCT |
| ITGA4_SBR | TAC ACA CCC TTC CCG AAA ACC | AGA GGT AAC ATG CCT GGC AGA |
| JUNB_SBR | GAC AGA CCA GAC CCT CAA GCA | TGC GTA TTT TTG ACC AGT GGA T |
| KANK4_SBR | GCT TTC GAG GAC AGC AGT CTG | GGG TGT GGA GAC AGC ACC TT |
| KLF7_SBR | CAG ATT CCC TTC TCT AAC CTG TCT AGA | AAC CAA AAT CTT CTG AAA TAG CCT TC |
| LAMB3_SBR | TGG CTG AGT ATG GAG AAA TAA GCA | GGT GGT GTT GAT TCC AGA CAA G |
| MRAP2_SBR | TGT ATC CAC CCT GAC CCT CC | CGC ATT GTC CTG GGC C |
| PADI4_SBR | TTG CAA TCA ACT GGA GCA GG | AGA GGG CTG AGG CCA CCT |
| PAWR_SBR | AAA TAC CCA CGT GTC TCT TTT CCT | CTG CCC GCT GCC CTA GT |
| PRR16_SBR | CCT GCC AAC TAC AAG CTG AAC A | GAT TAG CCT TGT CTG AGA CTG TGT CT |
| PTPN11_SBR | TTG TTC CTG GTG TCT GCT CTT C | AAC CCC CAG CCC CGT |
| S100A2_SBR | TCA GCC TCA CCC CCA CAT | TGT GGG CGA GAC CCT TAC C |
| SERPINE1_SBR | CAA CCT CAG CCA GAC AAG GT | CAG CCA CGT GAT TGT CTA GG |
| SLC7A8_SBR | TTT GGC TTG TTC TGT CAA AGA AAA | GAA GAC TTG CCC ATT GAG ACAAC |
| SMAD7_SBR | TTT TAA AGC GAC AGG GTG TCT AGA | TCT GCT CGG CTG GTT CCA |
| TLN2_SBR | GCT GGG CAC AGT GAC AAT GA | GCT TCC TCT GTG AGA CTT GTG ATT AC |
