# Supplementary material for: An integrated genomic approach identifies persistent tumor suppressive effects of transforming growth factor-β in human breast cancer
Source: Breast Cancer Res. 2014 Jun 2;16(3):R57. doi: 10.1186/bcr3668 (PMC4095608; doi:10.1186/bcr3668)
Supplement: Additional file 3 — Primer pairs for RT-QPCR. All primers are in 5′ to 3′ orientation. [file bcr3668-S3.docx]

**Additional file 3:** **Primer pairs for RTQ-PCR** All primers are in 5’ to 3’ orientation

| **TARGET** | **FORWARD PRIMER** | **REVERSE PRIMER** |
| --- | --- | --- |
| ANGPTL4_cDNA | GAC CCT GAG GTC CTT CAC AG | CCA CCT TGT GGA AGA GTT GC |
| ANXA2_cDNA | TGG CCC TGC TTT CAA CTG A | AGG AAG AAA GCT CTG GGA CTG A |
| C12ORF5_cDNA | GGG AAC ATG GCT CGC TTC | CCT TGG ATT ATT TTC TCC TTG TT |
| C15ORF57_cDNA | TGT GTC CGA GGA GAG AAG AAA | CTC ACG AGA GCA CGC AAA TA |
| CDH19_cDNA | GAT CTT AGC TGG CCG ATG TG | TGG GTG TGG AAC CAA TTT TT |
| COPA_cDNA | GGA GTC GGA GAC CTG AGA GA | CAA GGT CTT TTG GGG TGA AA |
| CTGF_cDNA | CAT CTT CGG TGG TAC GGT GT | AGG CAC GTG CAC TGG TAC TT |
| EFNA1_cDNA | CCA TGA CAA TCC ACA GGA GA | GCA CTG TGA CCG ATG CTA TG |
| FMNL2_cDNA | GGT GAA GAA TCC TCC CCA TA | GCC GTC TGA ATT TCT TCC TG |
| FRMD6_cDNA | TTA AGC TTC GTG GCC AGA GT | TGA TGT CAT CGA GGC TCA AG |
| FTSKD2_cDNA | TTT TGA CCT CCC TGC AGA CT | CCG GCC ATA GTA GCA AAT GT |
| IRF2BP2_cDNA | CCC TCT CCG TCC TCT ATG AA | CTG GTT GCC AGA GAG GAG TC |
| ITGA4_cDNA | GCT TTT CGG TCT GAT TCT GC | GGT GGC TTA AAG AAG CGT CA |
| KLF7_cDNA | TGG CAT CGG CTA GTG TCT AA | ACT TTC TTC TGC GAG GCA AT |
| LAMB3_cDNA | GGA ATG CTT TCC ATC TCC AG | GCT GCA GCT CAG GGT AAT CT |
| LIMK2_cDNA | GGT GTT CAG AAT GCC AGG AT | CCC AAA CTT CCC CCA GTA GT |
| PALM2-AKAP2_cDNA | TGC AAG CCA TAG CAG AAA AA | TCT GCT CGT CAA GCT GTT GT |
| PPIA_cDNA | GTC AAC CCC ACC GTG TTC TT | CTG CTG TCT TTG GGA CCT TGT |
| PTPN3_cDNA | CCC ACT GGA TAT TGT CCG AAA | TGG CTT GAT GTC TGC ACC AT |
| PTPN11_cDNA | CCT GAT GCA ACA GCA GAA AA | GGA GAG GGT GAA AGT CCA CA |
| S100A10_cDNA | CCT CTG GCT GTG GAC AAA AT | AAG CTC TGG AAG CCC ACT TT |
| SERPINE1_cDNA | GCG CTG CAG AAA GTG AAG AT | GCG GGC TGA GAC TAT GAC AG |
| SERPINE2_cDNA | CTC GCC ATG GTG ATG AGA TA | GAG ACG ATG GCC TTG TTG AT |
| SPAG9_cDNA | GAA GAT GCA AGG CAA AAA GC | TGG GCT GTG GGA ATA TCA CT |
| TMEM88_cDNA | GTGCTAGGGACCATCTTGCT | GGAACTGAGAGTGGCAGAGG |
| TPK1_cDNA | TCC AGT CTG TGG CTC CTA CC | TTT GTT CCA AAG ATG ACG AAA A |
| ZC3H7A_cDNA | CGT GCT CTC GTG AGA AAT CTT | GGC TTC CGT GTA CTG GCT TA |

EFNA1 primers (Hs00348886_m1) were purchased from Invitrogen.
