# Supplementary material for: An integrated genomic approach identifies persistent tumor suppressive effects of transforming growth factor-β in human breast cancer
Source: Breast Cancer Res. 2014 Jun 2;16(3):R57. doi: 10.1186/bcr3668 (PMC4095608; doi:10.1186/bcr3668)

**Additional file 5.** **ChIP-QPCR validation of Smad3 target genes with different occupancy patterns between the four cell lines.**

(A) 25 Smad3 target genes identified by TAS analysis of ChIP-chip data using an FDR of 0.15 were validated by ChIP-QPCR across all four cell lines and the pattern of gene occupancy was compared between the two methods. The table gives the summary of the results. ChIP-QPCR peaks were scored positive if TGF-β-induced occupancy was significant (p<0.05), and >2-fold over untreated. 65/67 of the Smad3 binding regions identified by TAS from ChIP-chip in one or more of the cell lines were validated by QPCR. However, Q-PCR was more sensitive and identified an additional 11/33 instances of Smad3 binding in genomic regions that were called negative in one or more of the cell lines by TAS (see eg. Smad3 occupancy of *LAMB3* promoter in M1 and M2). Thus the experimentally-determined FDR was 14%, with the majority (85%) of the false calls by ChIP-chip being false negatives. IL31RA is included as an example of a gene that did not show Smad3 occupancy in the ChIP-chip analysis.

|  | VALIDATION OF ChIP-Chip by Q-PCR | | | | | | | |
| --- | --- | --- | --- | --- | --- | --- | --- | --- |
| **Gene** | **M1** | | **M2** | | **M3** | | **M4** | |
|  | 15% FDR | Q-PCR | 15% FDR | Q-PCR | 15% FDR | Q-PCR | 15% FDR | Q-PCR |
| **ABLIM3** | **+** | **+** | **+** | **+** | **+** | **+** | **+** | **+** |
| **ADRA1B** | **+** | **+** | **+** | **+** | **+** | **+** | **+** | **+** |
| **ANXA2** | **-** | **+** | **-** | **+** | **+** | **+** | **+** | **+** |
| **CLSTN1** | **+** | **+** | **+** | **+** | **+** | **+** | **+** | **+** |
| **EEF1E1** | **-** | **-** | **-** | **-** | **+** | **-** | **+** | **-** |
| **F3** | **-** | **+** | **-** | **+** | **+** | **+** | **+** | **+** |
| **FAP** | **+** | **+** | **+** | **+** | **+** | **+** | **+** | **+** |
| **GAB1** | **-** | **-** | **-** | **-** | **-** | **-** | **+** | **+** |
| **IFNK** | **+** | **+** | **+** | **+** | **-** | **-** | **-** | **-** |
| **IL24** | **+** | **+** | **+** | **+** | **-** | **+** | **-** | **-** |
| **ITGA2** | **+** | **+** | **+** | **+** | **+** | **+** | **+** | **+** |
| **ITGA4** | **-** | **-** | **-** | **-** | **+** | **+** | **+** | **+** |
| **JUNB** | **+** | **+** | **+** | **+** | **+** | **+** | **+** | **+** |
| **KANK4** | **+** | **+** | **+** | **+** | **+** | **+** | **+** | **+** |
| **KLF7** | **+** | **+** | **+** | **+** | **+** | **+** | **+** | **+** |
| **LAMB3** | **-** | **+** | **-** | **+** | **+** | **+** | **+** | **+** |
| **MRAP2** | **-** | **+** | **-** | **+** | **+** | **+** | **+** | **+** |
| **PADI4** | **-** | **-** | **-** | **-** | **+** | **+** | **+** | **+** |
| **PAWR** | **-** | **-** | **-** | **-** | **+** | **+** | **-** | **-** |
| **PRR16** | **-** | **-** | **-** | **+** | **+** | **+** | **+** | **+** |
| **PTPN11** | **-** | **-** | **-** | **-** | **+** | **+** | **-** | **-** |
| **S100A2** | **+** | **+** | **+** | **+** | **+** | **+** | **+** | **+** |
| **SERPINE1** | **+** | **+** | **+** | **+** | **+** | **+** | **+** | **+** |
| **SLC7A8** | **+** | **+** | **+** | **+** | **+** | **+** | **-** | **-** |
| **TLN2** | **-** | **-** | **-** | **-** | **+** | **+** | **-** | **+** |
| **IL31RA** | **-** | **-** | **-** | **-** | **-** | **-** | **-** | **-** |

**(B) See next page**

(B) Representative ChIP-QPCR validation results are given for genes that show different patterns of Smad3 promoter occupancy between the four cell lines by ChIP-chip. Results are mean +/- SEM for 3 replicates. * Smad3 occupancy was induced >2-fold by TGF-β and was statistically significant (P<0.05; unpaired t-test). *IL31RA* was selected as a gene that did not show Smad3 binding by ChIP-chip. αS3, anti-Smad3 antibody; CON, control IgG.


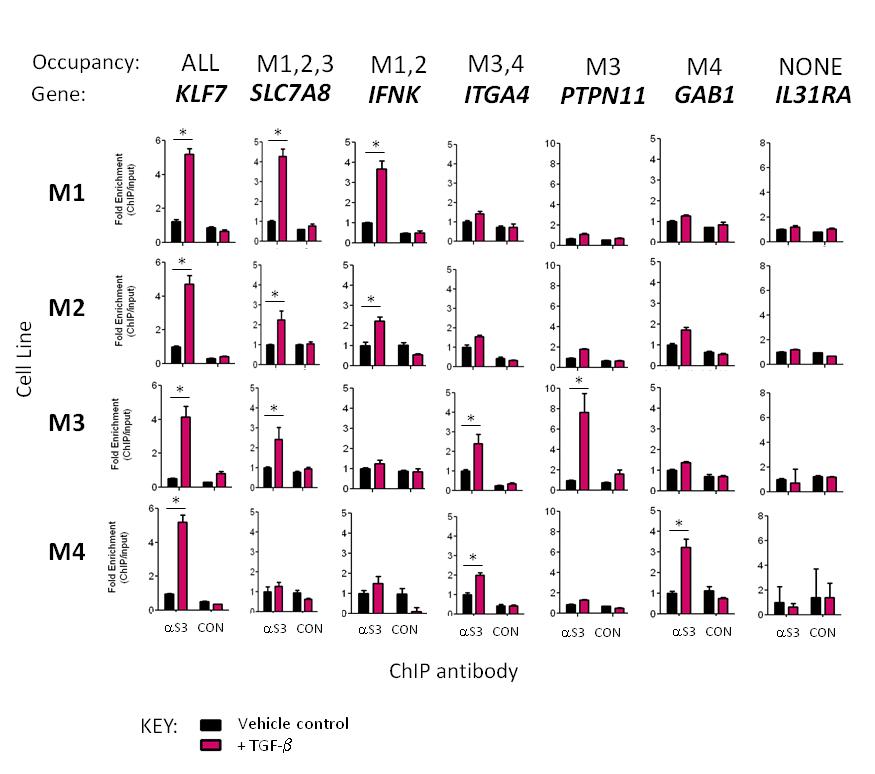

Supplement: Additional file 5 — ChIP-QPCR validation of Smad3 target genes with different occupancy patterns between the four cell lines. (A) A total of 25 Smad3 target genes identified by TAS analysis of ChIP-chip data using an FDR of 0.15 were validated by ChIP-QPCR across all four cell lines and the pattern of gene occupancy was compared between the two methods. The table gives the summary of the results. ChIP-QPCR peaks were scored positive if TGF-β-induced occupancy was significant (P <0.05), and ≥2-fold over untreated. A total of 65/67 of the Smad3 binding regions identified by TAS from ChIP-chip in one or more of the cell lines were validated by QPCR. However, QPCR was more sensitive and identified an additional 11/33 instances of Smad3 binding in genomic regions that were called negative in one or more of the cell lines by TAS (see for example Smad3 occupancy of LAMB3 promoter in M1 and M2). Thus the experimentally determined FDR was 14%, with the majority (85%) of the false calls by ChIP-chip being false negatives. IL31RA is included as an example of a gene that did not show Smad3 occupancy in the ChIP-chip analysis. (B) Representative ChIP-QPCR validation results are given for genes that show different patterns of Smad3 promoter occupancy between the four cell lines by ChIP-chip. Results are mean +/−SEM for three replicates. *Smad3 occupancy was induced ≥2-fold by TGF-β and was statistically significant (P <0.05; unpaired t test). IL31RA was selected as a gene that did not show Smad3 binding by ChIP-chip. αS3, anti-Smad3 antibody; CON, control IgG. [file bcr3668-S5.docx]
