# Supplementary material for: An integrated genomic approach identifies persistent tumor suppressive effects of transforming growth factor-β in human breast cancer
Source: Breast Cancer Res. 2014 Jun 2;16(3):R57. doi: 10.1186/bcr3668 (PMC4095608; doi:10.1186/bcr3668)
Supplement: Additional file 6 — Petal plot showing patterns of TGF-β-regulated gene expression in M1 to M4 cells. Global TGF-β-regulated gene expression was determined by microarray analysis at the 6 hour time point for all four cell lines. Using a fold-change cutoff of 1.5x and a significance cutoff of P = 0.001, a total of 563 genes were found to be significantly changed in their expression across the four cell lines. The majority of these genes were unique to the individual cell lines. [file bcr3668-S6.docx]

**Additional file 6: Petal plot showing patterns of TGF-β-regulated gene expression in M1-M4 cells.** Global TGF-β-regulated gene expression was determined by microarray analysis at the 6 hour timepoint for all four cell lines. Using a fold-change cutoff of 1.5x and a significance cutoff of p=0.001, a total of 563 genes were found to be significantly changed in their expression across the four cell lines. The majority of these genes were unique to the individual cell lines. In each segment of the petal plot, the upper numeral indicates the number of genes that were regulated and below that the cell line combination in which these genes were expressed is given.

**
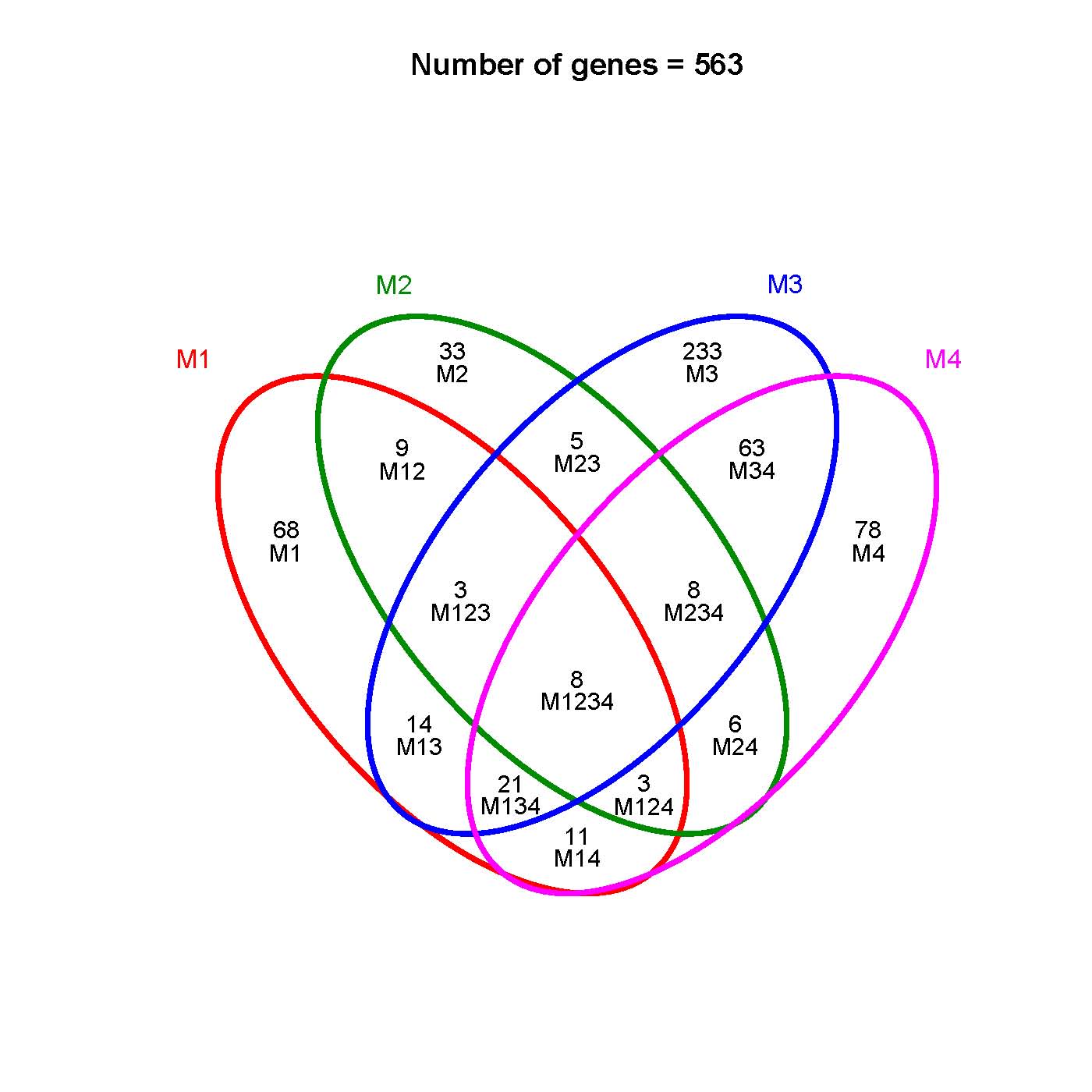
**
