# Supplementary material for: An integrated genomic approach identifies persistent tumor suppressive effects of transforming growth factor-β in human breast cancer
Source: Breast Cancer Res. 2014 Jun 2;16(3):R57. doi: 10.1186/bcr3668 (PMC4095608; doi:10.1186/bcr3668)
Supplement: Additional file 7 — Expression of Smad3 occupied genes in vitro. Considering only those genes that showed TGF-β-induced Smad3 occupancy, the fraction of TGF-β/Smad3 target genes showing regulated mRNA expression at 1 hour or 6 hours was determined from the microarray analysis using a P value cutoff of <0.001 for differential expression between the TGF-β-treated and untreated condition for a given cell line and time point. SBR, Smad3 binding region. [file bcr3668-S7.docx]

**Additional file 7. Expression of Smad3 occupied genes *in vitro***

Considering only those genes that showed TGF-β-induced Smad3 occupancy, the fraction of TGF-β/Smad3 target genes showing regulated mRNA expression at 1h or 6h after TGF-β treatment *in vitro* was determined from the microarray analysis using a p-value cutoff of <0.001 for differential expression between the TGF-β-treated and untreated condition for a given cell line and timepoint. SBR, Smad3 binding region.

|  | # Genes with SBRs | **Differentially expressed Smad3 targets** | | |
| --- | --- | --- | --- | --- |
|  |  | 1h | 6h | 1h or 6h |
| Cell line |  | # (%) | # (%) | # (%) |
| M1 | 93 | 24 (26) | 38 (41) | 50 (54) |
| M2 | 79 | 16 (20) | 20 (38) | 36 (46) |
| M3 | 281 | 61 (22) | 131 (47) | 147 (52) |
| M4 | 231 | 57 (25) | 111 (48) | 127 (55) |
