# Supplementary material for: An integrated genomic approach identifies persistent tumor suppressive effects of transforming growth factor-β in human breast cancer
Source: Breast Cancer Res. 2014 Jun 2;16(3):R57. doi: 10.1186/bcr3668 (PMC4095608; doi:10.1186/bcr3668)
Supplement: Additional file 8 — Immunostaining of M3 tumors for ANGPTL4 and SERPINE1. M3 tumors were immunostained for ANGPTL4 and SERPINE1 as described in Methods. Immunostaining for both proteins was observed predominantly in the tumor parenchyma (T) and not in the stroma (S). Scale bar represents 25 μm. [file bcr3668-S8.docx]

**Additional file 8. Immunostaining of M3 tumors for ANGPTL4 and SERPINE1.**

M3 tumors were immunostained for ANGPTL4 and SERPINE1 as described in Methods. Immunostaining for both proteins was observed predominantly in the tumor parenchyma (T) and not in the stroma (S). Scale bar is 25 μm.
