# Supplementary material for: An integrated genomic approach identifies persistent tumor suppressive effects of transforming growth factor-β in human breast cancer
Source: Breast Cancer Res. 2014 Jun 2;16(3):R57. doi: 10.1186/bcr3668 (PMC4095608; doi:10.1186/bcr3668)
Supplement: Additional file 10 — Prognostic power of the in vitro/in vivo concordant and discordant genes sets from the TSTSS when analyzed separately. Kaplan-Meier survival curves and multivariate analyses were generated within the GOBO breast cancer datasets using (A) only genes whose direction of regulation by TGF-β was concordant between in vitro and in vivo; (B) only genes whose direction of regulation was discordant in vitro and in vivo; and (C) the full TSTSS which includes both gene sets. Of the 26 genes of the TSTSS, 20 were concordant and 6 were discordant. A total of 16 of the 20 concordant genes were found in the GOBO datasets (not found: ANXA2, C15orf57, FRMD6 and IRF2BP2). Four of the six discordant genes were found in the GOBO datasets (not found: FMNL2 and TMEM88). [file bcr3668-S10.docx]

**Additional file 10. Prognostic power of the *in vitro/in vivo* concordant and discordant genes sets from the TSTSS when analysed separately.** Kaplan-Meier survival curves and multivariate analyses were generated within the GOBO breast cancer datasets using (**A**) only genes whose direction of regulation by TGF-β was concordant between *in vitro* and *in vivo*; (**B**) only genes whose direction of regulation was discordant *in vitro* and *in vivo*; and (**C**) the full TSTSS which includes both gene sets. Of the 26 genes of the TSTSS, 20 were concordant and 6 were discordant. 16 of the 20 concordant genes were found in the GOBO datasets *(not found: ANXA2, C15orf57, FRMD6 and IRF2BP2)*. 4 of the 6 discordant genes were found in the GOBO datasets *(not found: FMNL2 and TMEM88)*.

**A. CONCORDANT GENES ONLY**

**B. DISCORDANT GENES ONLY**

**C. COMPLETE TSTSS INCLUDING CONCORDANT AND DISCORDANT GENES**
