# Supplementary material for: An integrated genomic approach identifies persistent tumor suppressive effects of transforming growth factor-β in human breast cancer
Source: Breast Cancer Res. 2014 Jun 2;16(3):R57. doi: 10.1186/bcr3668 (PMC4095608; doi:10.1186/bcr3668)

**Additional file 11: Performance of a “generic” TGF-β signature in breast cancer cohorts using the GOBO metaanalysis tool.** Kaplan-Meier analyses were performed using the online GOBO tool to assess the association of the TGF-β-regulated gene-sets with distant metastasis-free survival (DMFS) in a meta-analysis across the multiple breast cancer cohorts of the GOBO dataset (1379 tumors from 8 cohorts). Patient datasets representing all tumors were dichotomized to higher than median expression (black) or lower than median expression (grey) of the geneset. P-values were determined by the Log-rank test. This figure shows Kaplan Meier plots for survival of all patients in the GOBO datasets, using a “generic TGF-β signature derived from the set of TGF-β/Smad3 target genes that were regulated in both M3 and M4 cells, and thus not enriched for tumor suppressor activity. Weighting (positive or negative) of individual target genes is based on the directionality of TGF-β regulated expression of this geneset in M3 cells *in vitro* **(A)** or in M3 tumors *in vivo* **(B)** as indicated. The list of genes involved is given in **Additional file 9**. Note the greatly reduced statistical power of this signature when compared to the use of the TSTSS tumor suppressor signature in the same cohorts **(Fig 6A)**. Note also that high expression of the generic TGF-β signature is associated with either good or bad outcome depending on whether the *in vitro* or *in vivo* directional weighting was used, again illustrating the strong influence that the biological context in which the signature was generated has on its subsequent performance in the clinical datasets.


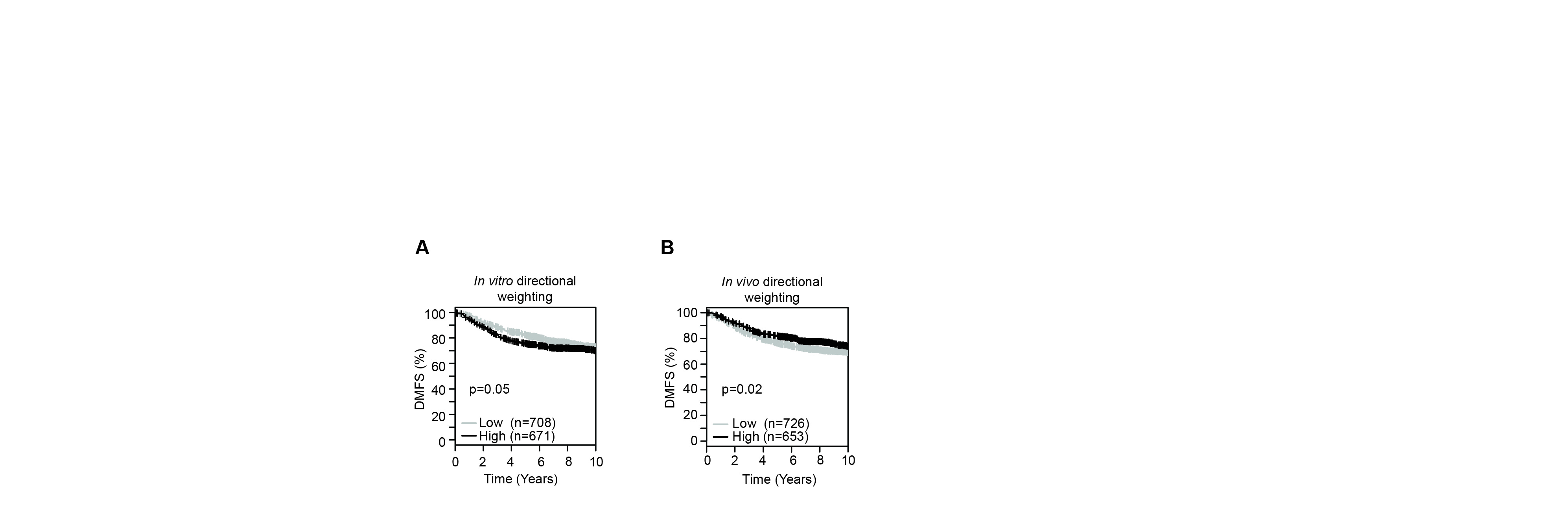

Supplement: Additional file 11 — Performance of a ‘generic’ TGF-β signature in breast cancer cohorts using the GOBO meta-analysis tool. Kaplan-Meier analyses were performed using the online GOBO tool to assess the association of the TGF-β-regulated gene sets with distant metastasis-free survival (DMFS) in a meta-analysis across the multiple breast cancer cohorts of the GOBO dataset (1,379 tumors from eight cohorts). Patient datasets representing all tumors were dichotomized to higher than median expression (black) or lower than median expression (grey) of the gene set. P values were determined by the log-rank test. This figure shows Kaplan-Meier plots for survival of all patients in the GOBO datasets, using a ‘generic’ TGF-β signature derived from the set of TGF-β/Smad3 target genes that were regulated in both M3 and M4 cells, and thus not enriched for tumor-suppressor activity. Weighting (positive or negative) of individual target genes is based on the directionality of TGF-β-regulated expression of this gene set in M3 cells in vitro(A) or in M3 tumors in vivo(B) as indicated. The list of genes involved is given in Additional file 9. Note the greatly reduced statistical power of this signature when compared to the use of the TSTSS tumor-suppressor signature in the same cohorts (Figure 6A). Note also that high expression of the generic TGF-β signature is associated with either good or bad outcome depending on whether the in vitro or in vivo directional weighting was used, again illustrating the strong influence that the biological context in which the signature was generated has on its subsequent performance in the clinical datasets. [file bcr3668-S11.docx]
