# Supplementary material for: An integrated genomic approach identifies persistent tumor suppressive effects of transforming growth factor-β in human breast cancer
Source: Breast Cancer Res. 2014 Jun 2;16(3):R57. doi: 10.1186/bcr3668 (PMC4095608; doi:10.1186/bcr3668)

**Additional file 12. Performance of the TSTSS in independent breast cancer cohorts** Kaplan-Meier analyses were performed using the R Package “Survival” to assess the association of the TGF-β-regulated gene-sets with distant metastasis-free survival (DMFS) or overall survival (OS) in three independent breast cancer datasets that were not included in the GOBO metaanalysis as they used different gene expression array platforms. Patient datasets were dichotomized to higher than median expression (black) or lower than median expression (grey) of the geneset. P-values were determined by the Log-rank test. Performance of the TSTSS (*in vivo* weighting) is shown for ER+ breast cancer datasets from **(A)** the Nederlands Kanker Instituut (NKI: n=249), **(B)** the TCGA cohort (n=407) and **(C)** the BT2000/Metabric (n=1508) cohorts. **(D)** GSE6532 (Loi) is a component dataset from the GOBO cohorts using the Affymetrix array platform that was reanalyzed using the same R Package method for direct comparison.


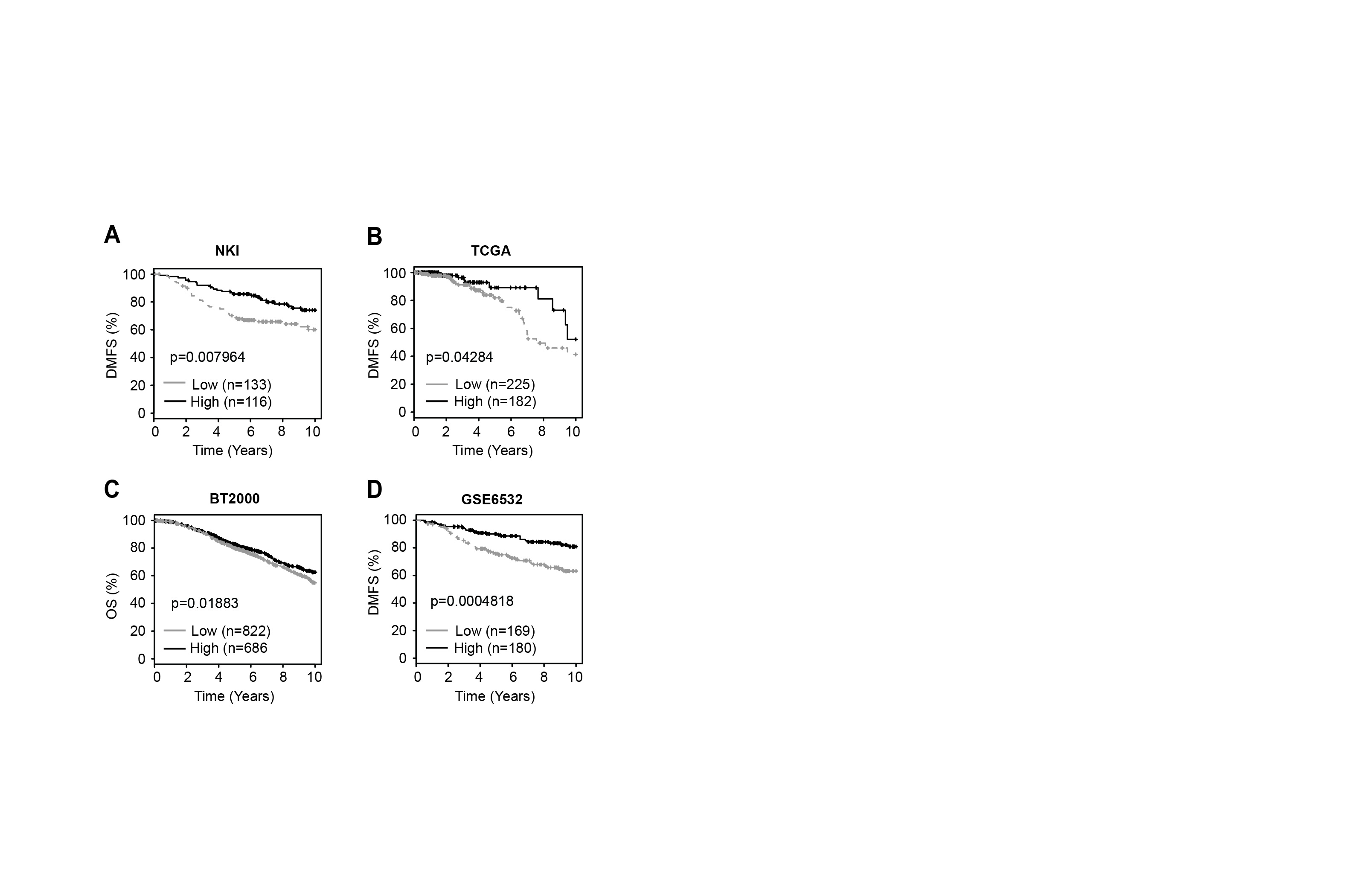

Supplement: Additional file 12 — Performance of the TSTSS in independent breast cancer cohorts. Kaplan-Meier analyses were performed using the R Package ‘Survival’ to assess the association of the TGF-β-regulated gene sets with distant metastasis-free survival (DMFS) or overall survival (OS) in three independent breast cancer datasets that were not included in the GOBO meta-analysis as they used different gene expression array platforms. Patient datasets were dichotomized to higher than median expression (black) or lower than median expression (grey) of the gene set. P values were determined by the log-rank test. Performance of the TSTSS (in vivo weighting) is shown for ER+ breast cancer datasets from (A) the Nederlands Kanker Instituut (NKI: n = 249), (B) the Cancer Genome Atlas (TCGA) cohort (n = 407) and (C) the BT2000/Metabric (n = 1508) cohorts. (D) GSE6532 (Loi) is a component dataset from the GOBO cohorts using the Affymetrix array platform that was reanalyzed using the same R Package method for direct comparison. [file bcr3668-S12.docx]
