# Supplementary material for: An integrated genomic approach identifies persistent tumor suppressive effects of transforming growth factor-β in human breast cancer
Source: Breast Cancer Res. 2014 Jun 2;16(3):R57. doi: 10.1186/bcr3668 (PMC4095608; doi:10.1186/bcr3668)
Supplement: Additional file 13 — Correlation between the metaPCNA index or the metaEphrin index and the TSTSS in additional ER+ breast cancer cohorts. The metaPCNA index (A) is a surrogate for proliferation and the metaEphrin index (B) is a surrogate for ephrin pathway activation in normal cells. More details on the indices are given in Methods. The GSE6532 (Loi) dataset contains 262 ER+ tumors, and the Nederlands Kanker Instituut (NKI) dataset contains 249 ER+ tumors. The Spearman correlation coefficient is given. [file bcr3668-S13.docx]

**Additional file 13**. **Correlation between the metaPCNA index or the metaEphrin index and the TSTSS in additional ER+ breast cancer cohorts.** The metaPCNA index **(A)** is a surrogate for proliferation and the metaEphrin index **(B)** is a surrogate for Ephrin pathway activation in normal cells. More details on the indices are given in **Methods.** The GSE6532 (Loi) dataset contains 262 ER+ tumors, and the Nederlands Kanker Institut (NKI) dataset contains 249 ER+ tumors. The Spearman correlation coefficient is given.
