# Supplementary material for: An integrated genomic approach identifies persistent tumor suppressive effects of transforming growth factor-β in human breast cancer
Source: Breast Cancer Res. 2014 Jun 2;16(3):R57. doi: 10.1186/bcr3668 (PMC4095608; doi:10.1186/bcr3668)
Supplement: Additional file 14 — Network analysis on the genes of the TSTSS. Ingenuity Pathway Analysis was performed to identify networks formed by the 26 genes of the TSTSS, and the top two networks are shown. Network 1 (score 32) is associated with the following network functions: Cardiovascular System Development and Function, Embryonic Development and Function. Network 2 (score 29) is associated with Cell Cycle, Digestive System Development and Function, and Cancer. Red indicates TGF-β/Smad3 target genes that were upregulated by TGF-β in vivo, and green indicates downregulated target genes. White shows non-target genes that were used to generate the networks. [file bcr3668-S14.docx]

**Additional file 14**. **Network analysis on the genes of the TSTSS.** Ingenuity Pathway Analysis was performed to identify networks formed by the 26 genes of the TSTSS, and the top two networks are shown. Network 1 (score 32) is associated with the following Network Functions: Cardiovascular System Development and Function, Embryonic Development and Function. Network 2 (score 29) is associated with Cell Cycle, Digestive System Development and Function, and Cancer. Red indicates TGF-β/Smad3 target genes that were upregulated by TGF-β *in vivo*, and green indicates down-regulated target genes. White shows non-target genes that were used to generate the networks.

**NETWORK 1**

**
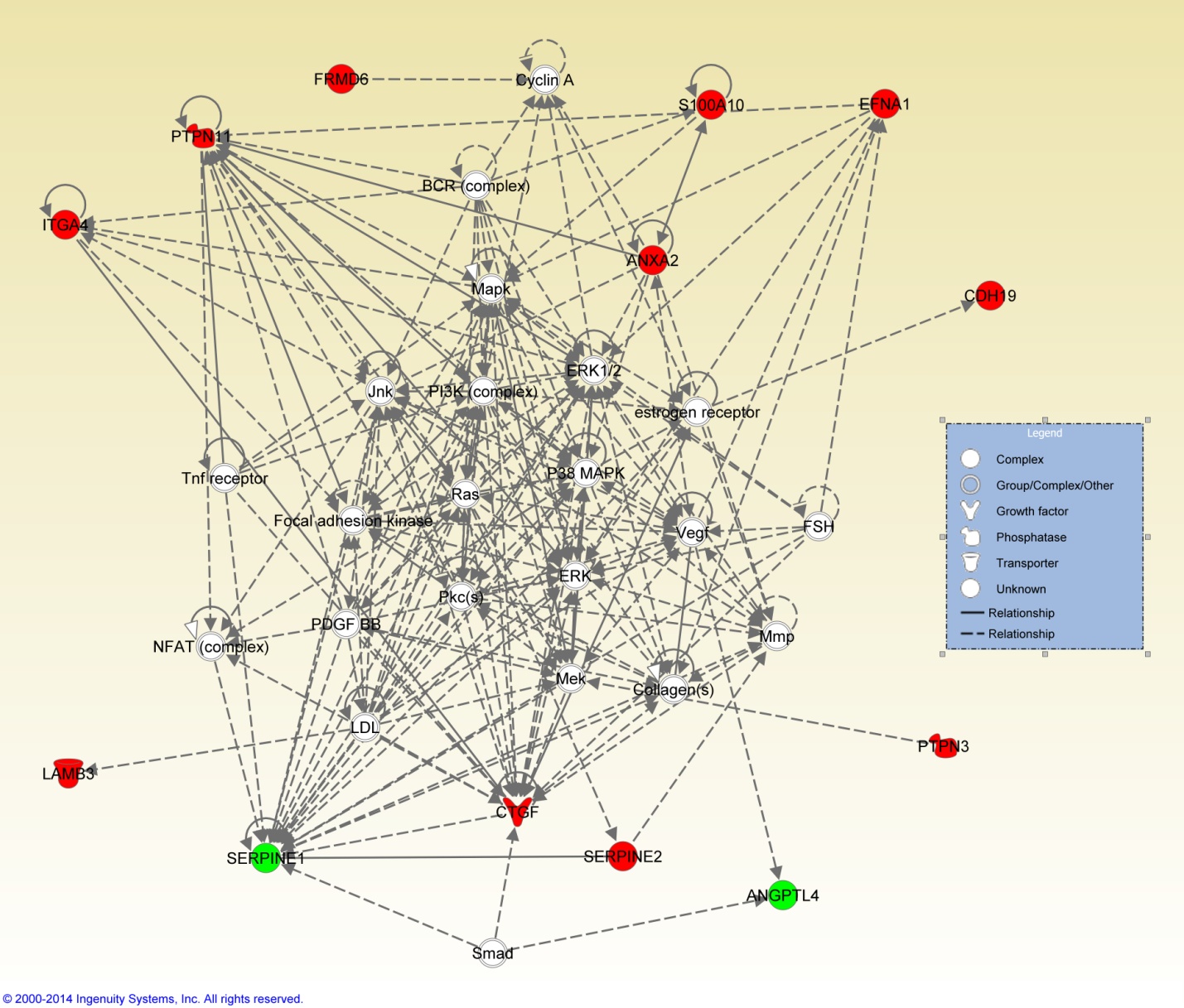
**

**NETWORK 2**

**
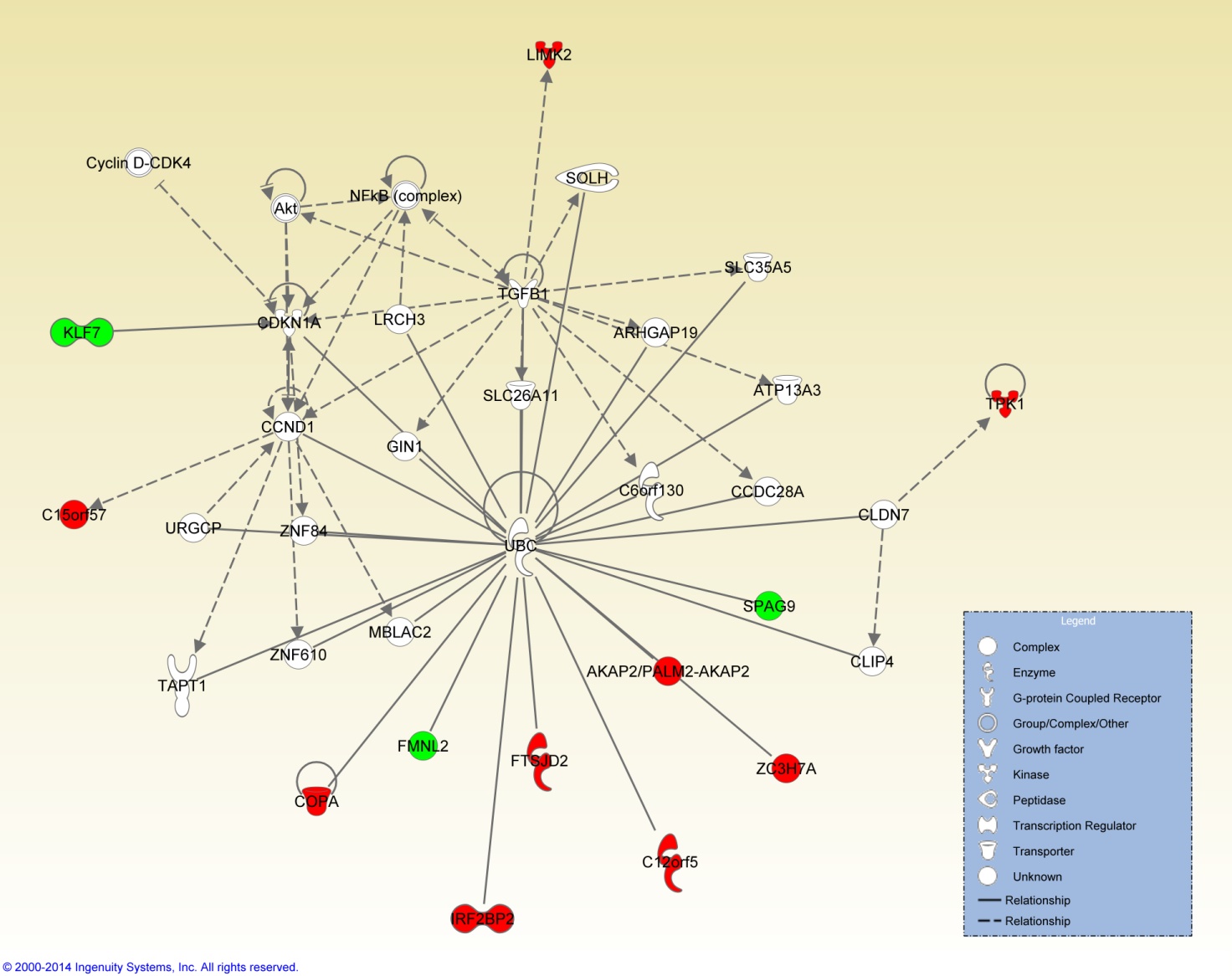
**
