# Supplementary material for: An integrated genomic approach identifies persistent tumor suppressive effects of transforming growth factor-β in human breast cancer
Source: Breast Cancer Res. 2014 Jun 2;16(3):R57. doi: 10.1186/bcr3668 (PMC4095608; doi:10.1186/bcr3668)
Supplement: Additional file 15 — Relative expression of the TSTSS in different human breast cancer subtypes. Analyses were done using the GOBO algorithm applied to all breast cancers in the GOBO database. (A) TSTSS expression in breast cancers stratified by ER status. (B) TSTSS expression in breast cancers stratified by intrinsic molecular subtype. HER2, HER2 amplified; Lum, luminal. The numbers of tumors in each category is given at the top of the figure. ANOVA P values. [file bcr3668-S15.docx]

**Additional file 15**. **Relative expression of the TSTSS in different human breast cancer subtypes.** Analyses were done using the GOBO algorithm applied to all breast cancers in the GOBO database. **A.** TSTSS expression in breast cancers stratified by ER status. **B.** TSTSS expression in breast cancers stratified by intrinsic molecular subtype. HER2, HER2 amplified; Lum, luminal. The numbers of tumors in each category is given at the top of the figure. ANOVA p-values.
